# Mechanical Parameters and Microstructural Evolution of FDM-Printed PLA and PLA+CF Under Variable Infill Architecture and Lubricant Exposure

**DOI:** 10.3390/polym18010072

**Published:** 2025-12-26

**Authors:** Emine Hozdić, Elvis Hozdić

**Affiliations:** Faculty of Mechanical Engineering, University of Novo Mesto, Na Loko 2, SI-8000 Novo Mesto, Slovenia; emine.hozdic@fs-unm.si

**Keywords:** Fused Deposition Modeling (FDM), PLA, PLA+CF, infill geometry, infill density, lubricant exposure, mechanical properties, microstructural analysis, porosity analysis

## Abstract

This study examines the influence of internal infill geometry, infill density, and short-term mineral oil exposure on the tensile and microstructural behavior of Fused Deposition Modeling (FDM) 3D-printed Polylactic Acid (PLA) and Carbon-Fiber-Reinforced PLA (PLA+CF). Standardized ISO 527-2 specimens were fabricated using linear, triangular, and hexagonal infill patterns at 30%, 60%, and 100% densities, followed by seven-day immersion in mineral oil. Mechanical testing and quantitative optical image analysis were performed to correlate porosity characteristics with tensile response. For PLA, the linear 30% infill achieved the highest tensile strength (31.5 MPa), while the hexagonal pattern exhibited the greatest ductility (ε = 4.9%). Oil exposure caused slight reductions in strength (−1.2%) and modulus (−4.1%) but increased elongation by 76%, indicating mild matrix plasticization. For PLA+CF, tensile strength and stiffness increased with density, reaching 33.4 MPa and 500 MPa at 100% infill, while oil exposure enhanced strength by 6.9% and reduced the average pore size from 475 µm^2^ to 146 µm^2^. Overall, the results demonstrate that optimizing infill topology, density, and fiber reinforcement significantly improves load transfer efficiency and environmental stability. These findings establish quantitative correlations between pore morphology and tensile behavior, providing a framework for the predictive design of environmentally resilient FDM polymer–composite components for semi-lubricated or tribological applications.

## 1. Introduction

The growing demand for high-performance, lightweight, and customizable components has intensified research into advanced polymeric and composite materials, particularly within the context of Additive Manufacturing (AM) technologies [1,2,3]. Among these, Fused Deposition Modeling (FDM)—also known as fused filament fabrication (FFF)—has emerged as one of the most widely adopted processes due to its cost efficiency, material versatility, and ability to produce geometrically complex structures [2]. The continued improvement of polymer feed stocks and printing systems has expanded the industrial relevance of FDM from rapid prototyping to the fabrication of functional components with tailored mechanical and structural properties.

In additive manufacturing, material selection requires a deep understanding of the interplay between functionality, process parameters, and design constraints, as these factors jointly determine the mechanical response and microstructural integrity of printed parts. The mechanical performance of FDM products depends not only on the intrinsic properties of the polymer or composite but also on process-induced features such as interlayer adhesion, porosity, and residual stresses [4]. Therefore, correlating microstructural evolution with mechanical behavior is essential for optimizing printing parameters and ensuring consistent performance in engineering applications.

AM provides clear advantages over conventional subtractive and formative methods such as casting, forging, and milling [5,6]. Its layer-by-layer deposition mechanism enables the fabrication of complex geometries with minimal waste, shorter production cycles, and high design flexibility [7,8]. Despite these advantages, FDM parts often exhibit inherent limitations such as anisotropic mechanical behavior, interlayer adhesion defects, and porosity, which can compromise their structural reliability and long-term performance. According to ASTM Committee F42, AM is defined as the process of constructing three-dimensional objects by sequential material addition [9]. ISO/ASTM 52900:2021 [10] categorizes AM technologies into several main groups, among which material extrusion—realized through FDM—remains the most accessible and industrially relevant method [11,12].

In the FDM process, a thermoplastic filament is heated to a molten state and extruded through a nozzle, forming successive layers that solidify to create the final part [13,14,15,16,17]. This simple yet effective principle enables accurate and repeatable fabrication of customized components at low cost. Due to its versatility and open accessibility, FDM is now widely used for both rapid prototyping and the production of functional end-use parts. Its ability to modify digital models and adjust material or process parameters without hardware changes makes it a key enabler of innovation in engineering design and product development [18,19,20,21].

Typical thermoplastic materials used in FDM include Polylactic Acid (PLA), Acrylonitrile Butadiene Styrene (ABS), and Polyethylene Terephthalate Glycol (PETG), valued for their processability, mechanical strength, and affordability [22,23,24]. More recently, composite filaments reinforced with short Carbon Fibers (CF), Glass Fibers (GF), or metallic fillers have been developed to improve stiffness, strength, and thermal stability [25,26,27,28]. The appropriate material choice and parameter tuning directly affect key mechanical properties—such as tensile strength, modulus, impact resistance, and ductility—relevant to the functional requirements of printed parts [29,30,31,32].

One of the distinctive features of FDM lies in its ability to create internal infill structures with adjustable geometry, density, and orientation, allowing mechanical performance to be tailored for specific loading scenarios. Common infill patterns—such as rectilinear, triangular, and hexagonal configurations—provide different trade-offs between stiffness, strength, and production efficiency [33,34,35,36]. Properly optimized infill structures can reduce material consumption and printing time while maintaining sufficient strength for engineering applications. However, both the infill design and process-induced microstructural features—such as voids, imperfect fusion, or misalignment between layers—play a decisive role in determining overall mechanical performance [37,38,39,40,41,42].

Numerous studies have examined how process parameters, infill design, and environmental conditions influence the mechanical and structural integrity of FDM-printed materials, demonstrating that layer thickness, printing speed, raster angle, and temperature strongly affect tensile performance and ductility, while external factors such as humidity, thermal cycling, or exposure to lubricants can promote microstructural degradation and delamination [43,44,45,46,47,48,49,50,51,52,53,54,55,56,57].

Recent tribological investigations have further confirmed that contact with lubricating fluids accelerates surface degradation and interfacial weakening in PLA- and PETG-based materials, particularly under repeated mechanical loading [54,58,59,60].

The effects of oil immersion on FDM-printed polymer materials have been investigated in detail in the authors’ previous work [59]. In the present study, lubricant exposure is therefore considered as a representative environmental condition integrated into a broader investigation of infill architecture, infill density, and material composition, rather than as a standalone oil immersion study.

In real service conditions, the combined effect of mechanical loading and environmental exposure—such as contact with lubricants, moisture, or temperature fluctuations—can accelerate material degradation and significantly alter the microstructural and mechanical stability of FDM-printed components. This interrelationship has been increasingly recognized as a key factor affecting the long-term reliability of additively manufactured materials.

Recent investigations have provided valuable insights into these relationships. Pascual-González et al. [61] demonstrated that heat treatment and improved interlayer adhesion enhance the microstructural integrity of carbon-fiber-reinforced composites. Guessasma et al. [62] reported that microstructural morphology significantly influences the tensile behavior of FDM biopolymers. Özen et al. [63] and Shanmugam et al. [64] observed that optimized processing conditions and homogeneous layer bonding improve both tensile and fatigue performance. Pandžić et al. [65] identified printing speed, temperature, and layer thickness as key determinants of strength in PLA and PLA+CF materials. Li et al. [66] and Zhen et al. [67] confirmed that controlled thermal treatment enhances the crystallinity and mechanical stability of PEEK. Patanwala et al. [68] and Naveed [69] found that nanoparticle reinforcement in PLA improves stiffness and tensile strength, while Kumar et al. [70] emphasized the role of fiber orientation and distribution in optimizing composite behavior.

Collectively, these studies demonstrate a strong interdependence between process parameters, material composition, and microstructural development in FDM-fabricated components. Yet, few studies have systematically explored how combined mechanical loading and environmental exposure—particularly contact with lubricants and moisture—alter the microstructural features and mechanical performance of FDM parts. This gap limits our understanding of structure–property relationships and the long-term durability of FDM materials in practical applications.

The novelty of this work lies in a systematic and quantitative investigation of the coupled effects of infill architecture, infill density, and lubricant exposure on both the mechanical response and microstructural evolution of FDM-printed PLA and PLA+CF materials. Unlike most existing studies that address these parameters independently, the present study establishes direct correlations between pore morphology (size, distribution, and total porosity) and tensile behavior using quantitative image analysis. Furthermore, the comparative evaluation of neat PLA and carbon-fiber-reinforced PLA under identical printing and environmental conditions provides new insights into the role of fiber reinforcement in enhancing environmental stability and load transfer efficiency in semi-lubricated service conditions.

The present study aims to bridge this gap by examining the correlation between microstructural characteristics and mechanical response in FDM-printed polymers and composites. Two materials—PLA and PLA+CF—were selected to represent both pure and fiber-reinforced polymers. To test this hypothesis, tensile testing (ISO 527-2) [71] was combined with optical microscopy to analyze layer cohesion, porosity, and fracture morphology before and after loading. It is hypothesized that optimized infill geometry and carbon fiber reinforcement improve layer cohesion and reduce microvoid formation, thereby enhancing tensile performance and resistance to environmental degradation.

This dual focus enables a deeper understanding of both fundamental material behavior and practical durability under real-world operating conditions.

From an industrial perspective, understanding the interplay between internal architecture, microstructure, and mechanical performance is essential for advancing the use of FDM materials in functional components operating under exposure to lubricants, oils, or variable temperatures. The findings of this study will contribute to a better understanding of the reliability of FDM-manufactured components frequently exposed to such environmental factors—such as housings, brackets, and load-bearing elements in machinery.

Overall, the introduction of this research framework provides both scientific and practical insights for optimizing FDM-printed polymer and composite components used in demanding industrial environments.

This study hypothesizes that both the internal infill architecture and exposure to lubricating oils synergistically influence the tensile and microstructural performance of FDM-printed PLA and PLA+CF materials. The objectives are to quantify these effects through standardized tensile testing, microstructural image analysis, and correlation of porosity metrics with mechanical response.

## 2. Materials and Methods

### 2.1. Material Specification for 3D Printing

Two polymeric filaments—Polylactic Acid (PLA) and PLA reinforced with short carbon fibers (PLA+CF)—were used to fabricate the tensile test specimens. Both materials were commercial 1.75 mm filaments supplied by Zhejiang FlashForge 3D Technology Co., Ltd. (Zhejiang, China) [72]. The PLA+CF filament used in this study is a commercially available material containing short, randomly oriented carbon fibers dispersed in the PLA matrix; detailed information on fiber length, diameter, and hardness is not disclosed by the manufacturer.

The addition of short carbon fibers (average length ≈ 100 µm) enhances stiffness, heat resistance, and interlayer bonding while reducing shrinkage and residual stresses during solidification. The selection of both pure and composite filaments enabled a comparative analysis of the effect of carbon-fiber reinforcement on the mechanical and microstructural response of FDM-printed components under identical process conditions.

Table 1 and Table 2 present the key physical and mechanical properties of the materials, as reported by the manufacturer and verified by the tensile tests conducted in this study. Including this table provides a clear baseline for the comparative analysis of PLA and its CF-reinforced counterparts, highlighting expected differences in stiffness and ductility prior to experimental evaluation.

### 2.2. Preparation and Printing of Tensile Test Specimens

The tensile specimens were designed in accordance with the ISO 527-2:2012 standard [71]. The 3D models were created using SolidWorks 2023 (Dassault Systèmes, Paris, France) and subsequently sliced in FlashPrint5 software 5 (http://www.flashforge.com/ accessed on 18 August 2023). Fabrication was performed on an Adventurer 4 Series 3D printer, manufactured by Zhejiang FlashForge 3D Technology Co., Ltd. [Zhejiang, China], with a 0.4 mm hardened steel nozzle and an automatic filament calibration system.

**Figure 1:**
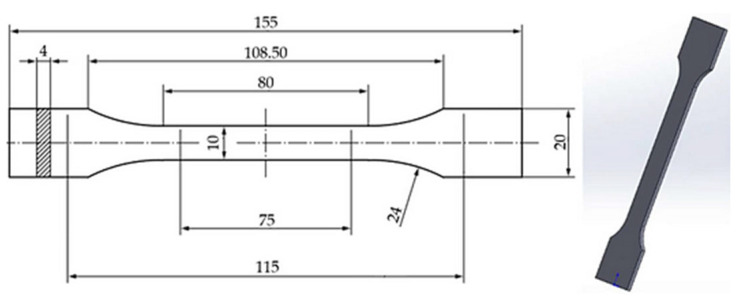
SolidWorks-generated model of standardized tensile specimen geometry (ISO 527-2: 2012) [71,73].

Figure 1 illustrates the CAD model of the standardized tensile specimen designed in SolidWorks, with dimensions defined by ISO 527-2 geometry [71]. The inclusion of this figure ensures replicability and provides a visual reference for the specimen configuration used across all tested materials.

**Figure 2:**
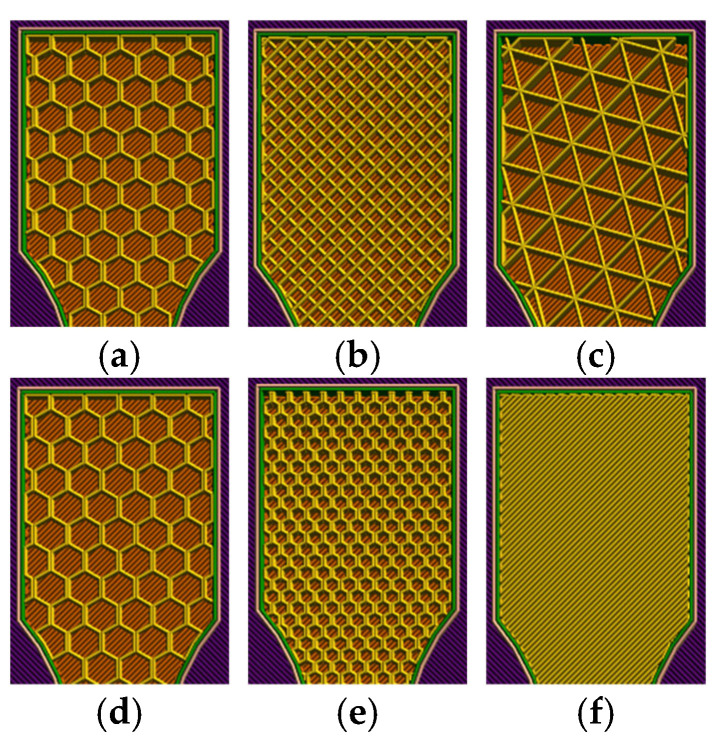
Representative slicer-generated models of FDM-printed PLA specimens used in three experimental series: (**a**–**c**) geometry series with 30% infill showing (**a**) linear, (**b**) triangular, and (**c**) hexagonal internal structures; (**d**–**f**) density series with linear infill at 30%, 60%, and 100% infill levels.

To systematically isolate the effect of internal architecture, three experimental series were fabricated under identical printer settings (nozzle Ø 0.40 mm, layer height 0.20 mm; first layer 0.30 mm; four top and three bottom layers; print/travel speeds 60/100 mm·s^−1^; bed 50 °C; extrusion 210 °C for PLA and 225 °C for PLA+CF). (i) Geometry series (30% infill): PLA specimens with three infill patterns—linear/rectilinear (raster 0° parallel to the tensile axis), triangular (slicer default equiangular toolpaths, nominal 0°/±60°), and hexagonal (slicer-generated honeycomb with equiangular deposition);, see Figure 2a–c. (ii) Density series (linear infill): PLA specimens with 30%, 60% and 100% infill, raster 0° to the tensile axis;, see Figure 2d–f. (iii) Oil-exposure series: PLA specimens with hexagonal, 30% infill printed as in the geometry series; one group remained unexposed, while the other was immersed 7 days in SAE 15W-40 (API SF/CD) prior to testing. For each condition, we prepared tensile coupons (suffix A) and companion, unfractured coupons for microscopy (suffix B), using specimen codes consistent with Section 3. After exposure, specimens were gently wiped and conditioned for 24 h prior to testing.

For all infill patterns, the infill density and extrusion parameters were kept constant; infill line spacing was automatically defined by the slicing software, while specimen mass and printing time were not evaluated, as the focus was placed on mechanical performance.

To achieve dimensional stability and eliminate residual moisture effects, all printed specimens were conditioned for 48 h in a controlled environment (23 ± 2 °C, 50 ± 5% RH) before testing.

Before mechanical testing, all specimens were visually inspected and measured using a digital caliper to verify dimensional conformity with the ISO 527-2:2012 standard [72]. This step ensured that deviations in mechanical results were not caused by geometric inaccuracies or printing defects.

This controlled and repeatable fabrication process ensured that any variations in measured mechanical performance could be attributed primarily to material composition and environmental exposure rather than inconsistencies in printing parameters.

Prior to printing, both PLA and PLA+CF filaments were dried in a ventilated oven at 50 °C for 8 h to minimize the effect of absorbed moisture on extrusion stability and interlayer adhesion. This step was necessary because PLA is known to be hygroscopic, and residual humidity can lead to hydrolytic degradation and bubble formation during melting. The controlled pre-drying ensured consistent filament feeding, uniform layer deposition, and reliable mechanical performance of the printed specimens.

### 2.3. Tensile Testing for 3D-Printed Specimens

Tensile testing was performed in accordance with ISO 527-2:2012 standard [72]. Mechanical characterization was conducted using a Shimadzu AGS-X universal testing machine (Kyoto, Japan) equipped with a 10 kN load cell and an integrated TrapeziumX control and data acquisition system.

The specimens were clamped using precision wedge grips to ensure uniform load distribution and minimize slippage. The tests were carried out under quasi-static loading at a constant crosshead speed of 5 mm/min and standard laboratory conditions (23 ± 2 °C, 50 ± 5% RH). Force and elongation were continuously measured by an extensometer with an accuracy of ±0.5%, allowing for precise determination of strain behavior throughout the test. The testing order of samples was randomized to minimize potential systematic bias.

The mineral oil used for immersion testing was a commercially available multigrade engine lubricant (SAE 15W-40, API SN/CF classification). The oil temperature during the seven-day exposure was maintained at 25 ± 2 °C in sealed glass containers to prevent evaporation and contamination. The specimens were fully submerged and subsequently dried at room temperature for 24 h before testing to ensure stable mass and surface conditions.

Specimens were tested for both material type (PLA and PLA+CF) and condition (unexposed and lubricant-exposed), providing a statistically relevant dataset. From the recorded load–displacement data, the following mechanical parameters were derived: tensile strength (σ), calculated as the maximum load divided by the initial cross-sectional area; Young’s modulus (E), obtained from the initial linear region of the stress–strain curve; nominal strain at break (εₑ), representing the percentage elongation at fracture; and maximum displacement (Δl), defined as the total elongation of the specimen at the point of failure.

For specimens with infill <100%, tensile metrics are reported as apparent values referenced to the gross cross-section (σ_app = F_max/A_gross; E_app from the initial slope of the apparent stress–strain curve). This convention preserves geometric comparability within the present framework but yields values that are lower than catalog properties obtained on fully dense, isotropic plastics. Where appropriate, we discuss how raster alignment, void topology and interlayer cohesion modulate these apparent properties.

For each experimental condition, three specimens (*n* = 3) were tested. The results are expressed as mean ± standard deviation (SD) and 95% confidence interval (CI). The normality of data distribution was verified using the Shapiro–Wilk test, while the homogeneity of variances was assessed with Levene’s test. Statistical comparisons between groups within each series (geometry, infill density, and oil exposure) were performed using one-way analysis of variance (ANOVA) followed by Tukey’s HSD post hoc test at a significance level of α = 0.05, or by the non-parametric Kruskal–Wallis test with Dunn’s multiple comparison correction where normality assumptions were not met. Effect sizes (η^2^) were calculated to evaluate the magnitude of differences, and potential outliers were screened using the robust median absolute deviation (MAD ± 3.5) criterion.

Given the small sample size (*n* = 3 per condition), normality and homoscedasticity tests have limited power, and effect-size estimates (η^2^) should be interpreted with caution. Accordingly, we report 95% confidence intervals and treat marginal differences as indicative trends rather than definitive effects.

### 2.4. Macroscopic and Microstructural Examination

Following 3D printing, environmental exposure (including the 7-day immersion in mineral engine oil), and tensile testing, all specimens underwent systematic preparation for macroscopic and microscopic examination. Each specimen was first labeled with an identification code, and its midsection was precisely marked to ensure consistency across all analyzed samples. The central portions were then sectioned into approximately 15 mm long segments using a precision band saw. These subsections represented characteristic regions of the specimens, preserving both fracture zones and undamaged areas necessary for comparative microstructural assessment.

**Figure 3:**
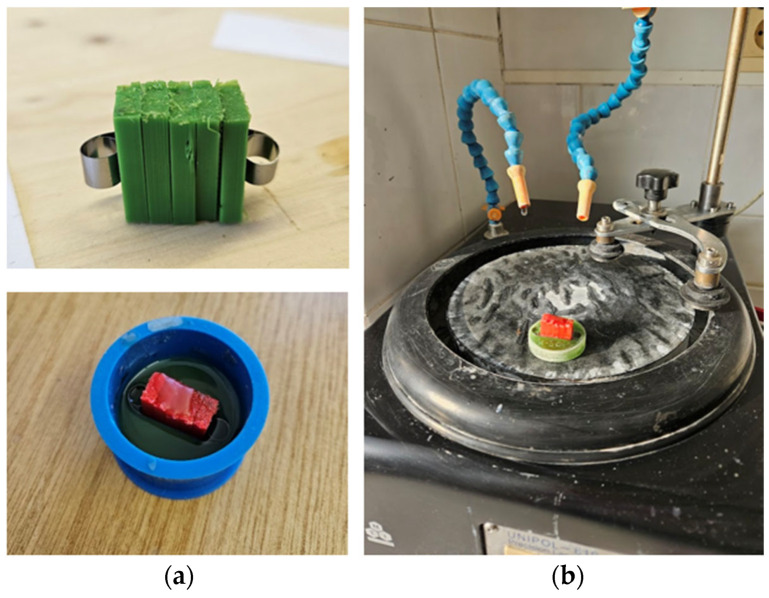
Sample preparation and surface finishing: (**a**) preparation and sectioning of specimens prior to embedding; (**b**) progressive surface grinding and polishing of mounted samples.

The extracted segments were subsequently embedded in epoxy resin molds to provide a stable base for surface preparation and analysis (Figure 3a). After complete resin curing, the mounted samples were subjected to progressive mechanical grinding on an MTI Corporation Unipol 810 grinding and polishing unit. The process began with coarse abrasive paper (P240) to remove excess resin and surface irregularities, followed by sequentially finer grades (P800, P1200, and P2500) to achieve a uniform and scratch-free surface finish (Figure 3b). Upon completion of grinding, all specimens were carefully rinsed, cleaned, and then polished using alumina particles with an average size of 0.05 µm to produce a mirror-like surface suitable for high-resolution microscopy. The same grinding and polishing protocol was applied to both PLA and PLA+CF specimens to ensure full comparability of the microstructural observations. The selected preparation sequence was chosen to minimize surface-induced artifacts, such as fiber pull-out or matrix smearing, and to preserve the intrinsic morphology of the FDM-printed structures. This ensured that the observed microstructural features reflect the material and process-related characteristics rather than preparation-related effects.

Macroscopic inspection of the prepared samples was first carried out using a VEVOR digital microscope (VEVOR, Ljubljana, Slovenia [74]), which provided a general overview of the surface morphology, identifying larger-scale defects such as delamination, void clusters, or surface tearing. This was followed by detailed optical microscopy performed with a Zeiss Axio Imager A1m (Carl Zeiss Microscopy, LLC, White Plains, NY, USA [75]). Image acquisition and documentation were conducted using the AxioVision software suite (Carl Zeiss AxioVision Rel. 4.8, White Plains, NY, USA [75]), ensuring precise calibration, focus uniformity, and reproducible imaging parameters across all specimens.

Subsequent quantitative image analysis was performed using Fiji (version 2.9.0) [76], an open-source platform based on ImageJ, which enabled accurate assessment of microstructural characteristics including pore size, shape distribution, and local heterogeneities in layer bonding. This digital image processing framework provided robust statistical data linking structural morphology with mechanical performance, particularly in identifying the initiation and propagation sites of cracks, the distribution of porosity, and the extent of interlayer cohesion.

Prior to quantitative evaluation, all microscopic images were calibrated in Fiji using a micrometric reference scale, where one division corresponded to 10 µm. This calibration step established the initial pixel-to-length conversion and ensured accurate spatial measurements across all magnifications. The scale bar displayed on the final figures (500 µm) reflects the actual magnification of the composite (stitched) images, which represent entire specimens with dimensions of approximately 40 × 100 mm. Each full-view image was composed of several overlapping microscopic subfields (individual FOV ≈ 2.5 × 1.9 mm), stitched together to achieve complete coverage of the gauge section while preserving micrometric accuracy.

Image preprocessing included background normalization and Gaussian smoothing (σ = 1.0) to minimize illumination gradients and improve segmentation contrast. These operations did not alter the specimen morphology but ensured consistent image quality and thresholding conditions across all analyzed regions. Segmentation of pores was performed using Otsu’s global thresholding (applied per image after background normalization). Post-threshold refinement used morphological opening and closing (disk kernel, radius = 2 px) to suppress isolated noise and merge fragmented pore boundaries.

Porosity (%) was computed as the ratio of pore pixels to analyzable pixels, multiplied by 100%. For each specimen, five randomly selected fields of view within the gauge section were analyzed; inter-field variability is reported as the coefficient of variation (CV, %). To mitigate edge bias, a 20-px guard band was applied along the image perimeter; pores intersecting this band were excluded, and the analyzable area was updated accordingly. After morphological cleaning, only pores with an equivalent area between 10 and 100,000 µm^2^ were retained for quantification.

This comprehensive preparation and analysis procedure ensured the reliability of both qualitative and quantitative observations, allowing a detailed correlation between mechanical performance, fracture morphology, and the internal structural features of FDM-printed PLA and PLA+CF specimens under varying geometric, density, and environmental conditions.

The microstructural analysis in this study was intentionally focused on evaluating porosity, interlayer bonding quality, and fracture morphology, as these features directly govern the tensile response of FDM-printed parts. The PLA+CF material contains short, discontinuous carbon fibers, whose individual visualization is limited when using optical microscopy. The influence of carbon fiber reinforcement is therefore assessed indirectly through its effect on the mechanical behavior of the material, primarily in terms of increased stiffness and tensile strength compared to neat PLA under identical processing conditions. This approach is consistent with existing studies on short-fiber-reinforced FDM filaments and enables a reliable correlation between microstructural characteristics and mechanical response.

## 3. Results

This section presents a comprehensive analysis of the microstructural changes observed in polymeric and composite materials fabricated using FDM technology, and their influence on the resulting mechanical properties. The study is based on tensile testing and microstructural characterization of specimens printed from two different materials: PLA, and PLA+CF. Each subsection of this chapter is structured into three integral parts that together provide a holistic understanding of how the selected process parameters affect the mechanical and structural performance of the tested materials. Section 3.1 reports PLA results; the PLA+CF composite is analyzed in Section 3.2 under identical protocols.

The first part of each subsection presents the results of tensile testing as a function of the infill structure geometry. Three distinct infill patterns—linear, triangular, and hexagonal—were analyzed, while maintaining a constant infill density of 30%. The obtained results include both mechanical parameters and microstructural features, elucidating the influence of the infill geometry on tensile strength, ductility, and internal structural integrity. Comparative analysis reveals how the geometric arrangement of the printed layers affects load distribution, crack propagation, and interlayer bonding, thereby determining the overall material performance under uniaxial stress.

The second part of each subsection focuses on the influence of infill density in specimens with a linear structure. Samples were printed at three infill densities—30%, 60%, and 100%—to evaluate the correlation between internal porosity and mechanical response. This section provides insight into how increasing the material content within the specimen influences stiffness, ultimate tensile strength, and failure mechanisms, as well as the corresponding microstructural features observed under optical microscopy. The results also serve as an experimental validation of the well-established relationship between the effective load-bearing cross-section and the apparent modulus of elasticity in FDM-printed polymers and composites.

The third part addresses the environmental effects on mechanical and microstructural behavior, with particular emphasis on exposure to mineral motor oil. In this stage of the experiment, samples with hexagonal infill geometry and 30% density were subjected to controlled oil exposure. The results demonstrate how environmental degradation—manifested through increased porosity, microcrack formation, and interlayer delamination—affects the mechanical performance of the materials, particularly their tensile strength. The findings highlight the susceptibility of polymeric and composite structures to chemical interactions with lubricants, providing critical information for potential applications in tribologically demanding environments.

Throughout this section, the results are presented using a combination of tables, graphs, and microscopic images, ensuring a clear visualization of the correlations between process parameters, microstructural morphology, and mechanical behavior. The primary objective of this chapter is to deliver a comprehensive and systematic presentation of the findings, forming a foundation for the optimization of FDM process parameters and the future development of polymeric and composite materials with enhanced structural integrity and environmental resistance.

### 3.1. Mechanical Parameters and Microstructural Response of 3D-Printed PLA Specimens

The mechanical behavior and microstructural response of FDM 3D-printed PLA specimens were systematically evaluated by examining the influence of infill structure geometry (Section 3.1.1), the infill density of the linear pattern (Section 3.1.2), and the duration of exposure to mineral motor oil in specimens with hexagonal infill structures (Section 3.1.3).

Across Section 3.1.1, Section 3.1.2 and Section 3.1.3, specimen codes are used as follows: geometry series (30% infill): V1A/V1B = hexagonal, V2A/V2B = triangular, V3A/V3B = linear. Density series (linear infill): V3A–V5A (tensile) and V3B–V5B (microstructure) correspond to 30%, 60%, 100%. Oil exposure (hexagonal, 30%): V1A/V1B = unexposed; V6A/V6B = 7-day exposure. Suffix A = tensile specimens; B = unfractured for microscopy.

#### 3.1.1. Influence of Infill Structure Geometry on the Mechanical and Microstructural Behavior of 3D-Printed PLA Specimens

The geometry of the internal infill structure plays a decisive role in determining the load-bearing capacity and microstructural integrity of FDM-printed polymer components. In particular, the arrangement of filament paths and the quality of interlayer bonding significantly influence the resulting mechanical performance.

**Figure 4:**
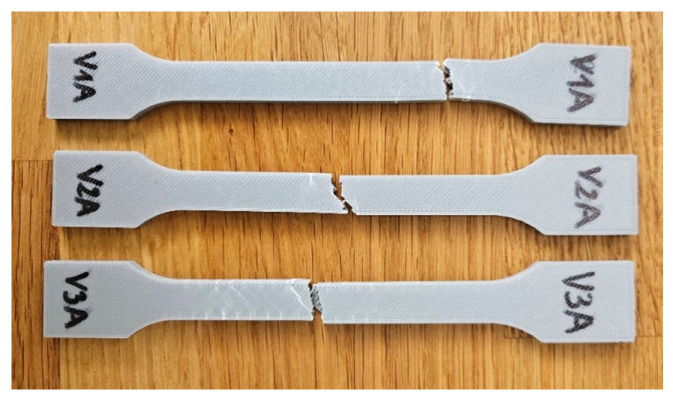
PLA tensile specimens printed with different infill geometries: hexagonal (V1A), triangular (V2A), and linear (V3A), all with 30% infill density. Macroscopic differences in fracture appearance reflect the effect of internal geometry on load-bearing behavior.

Figure 4 presents the PLA specimens after tensile testing, printed with three distinct infill geometries: hexagonal (V1A/PLA_211), triangular (V2A/PLA_222), and linear (V3A/PLA_232). All were printed with PLA and an infill density of 30%. The corresponding results of the tensile tests for all PLA specimens fabricated via FDM are summarized in Table 3. The comparative analysis of the principal mechanical parameters—tensile strength (σ), Young’s modulus (E), maximum displacement (Δl), and nominal strain at break (εₑ)—is shown in Figure 4.

**Figure 5:**
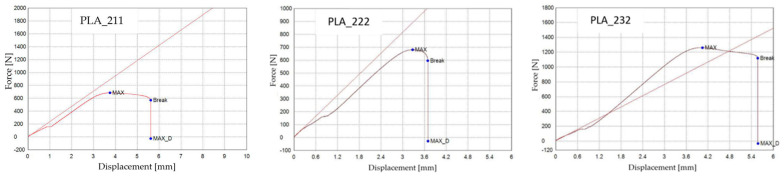
3D-printed PLA specimens tested: the force–displacement curves. The specimens PLA_211, PLA_222, and PLA_232 are representative samples of case V1A, V2A, and V3A, respectively.

**Figure 6:**
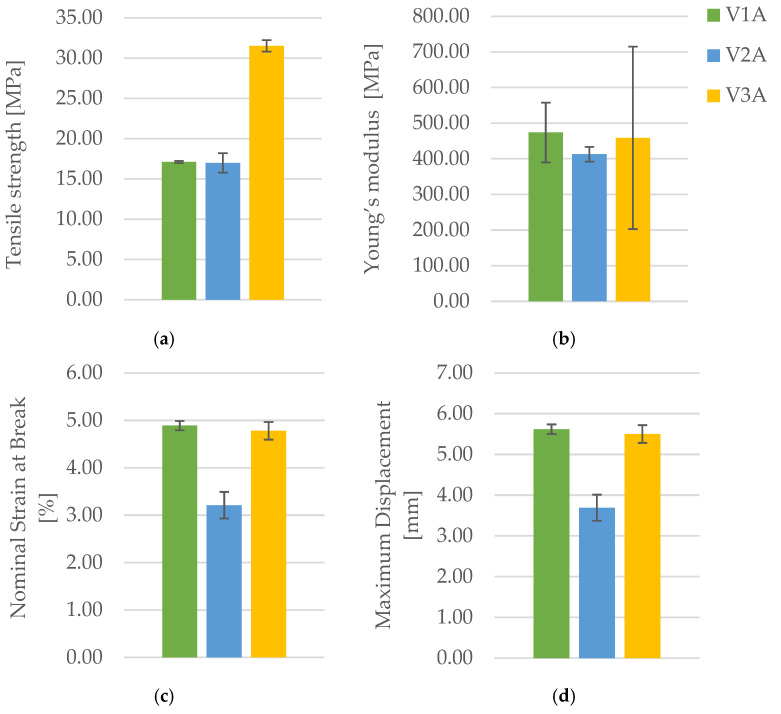
Comparison of tensile properties of PLA specimens with different infill geometries: (**a**) tensile strength, (**b**) Young’s modulus, (**c**) nominal strain at break, and (**d**) maximum displacement. The linear pattern (V3A/PLA_232) shows the highest tensile strength, while the hexagonal pattern (V1A/PLA_211) demonstrates superior ductility.

The results summarized in Table 3 and illustrated in Figure 5 and Figure 6 clearly demonstrate that infill geometry strongly influences the tensile behavior of FDM-printed PLA specimens.

Based on the analysis of the force–displacement curves presented in Figure 6 (the specimens PLA_211, PLA_222, and PLA_232 are representative samples of groups V1A, V2A, and V3A, respectively), it is evident that the 3D-printed PLA specimens exhibited a characteristic mechanical response under tensile loading, marked by an initial linear elastic region followed by progressive deformation up to fracture. Differences in the maximum force (F) and maximum displacement (Δl) among individual specimens indicate variability in stiffness and ductility, which can be attributed to the influence of internal infill architecture and process-induced microstructural features inherent to the FDM manufacturing process.

The linear infill pattern (V3A/PLA_232) exhibited the highest tensile strength (31.53 MPa) and moderate ductility (εₑ = 4.78%, Δl = 5.50 mm), indicating efficient stress transfer along the printing direction due to highly oriented filament paths. The hexagonal infill (V1A/PLA_211) showed lower tensile strength (17.10 MPa) but the highest elongation (εₑ = 4.89%, Δl = 5.62 mm), reflecting superior ductility and energy absorption capability. In contrast, the triangular infill (V2A/PLA_222) exhibited the lowest mechanical performance (σ = 16.99 MPa, E = 412.69 MPa, εₑ = 3.21%), attributed to stress concentration at the sharp cell junctions and weaker interlayer bonding, see Figure 6.

The linear infill was printed with raster lines aligned to the loading axis, enabling efficient axial load transfer and explaining the highest tensile strength in V3A/PLA_232. In contrast, hexagonal and especially triangular topologies introduce multi-directional paths and sharper junctions that elevate local stress concentration and reduce net load-bearing efficiency.

Overall, the linear pattern provided the highest tensile strength, while the hexagonal pattern displayed the most balanced performance, combining moderate stiffness with excellent ductility. The triangular pattern was mechanically the least favorable, reflecting more pronounced stress localization and limited deformation capacity prior to fracture.

The observed differences were further examined through a combined fractographic and microstructural approach to correlate macroscopic behavior with underlying structural features.

**Figure 7:**
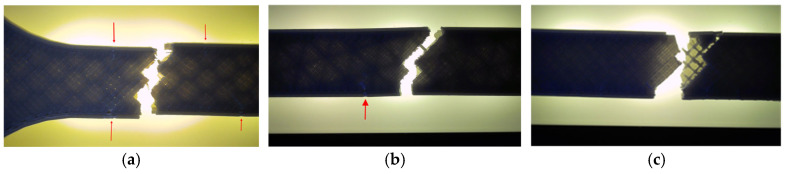
Fracture surfaces of PLA specimens after tensile testing: (**a**) V1A—hexagonal structure, (**b**) V2A—triangular structure, (**c**) V3A—linear structure. Visible stress concentration zones and crack propagation paths indicate distinct fracture mechanisms for each geometry.

Fractographic analysis provided further insight into the distinct failure mechanisms observed among the specimens. The hexagonal specimen (V1A/PLA_211) (Figure 7a) exhibited a clean and relatively brittle fracture at the narrowest cross-section, where stress concentration was highest. Subtle color changes along the internal walls, especially at the intersections of the hexagonal cells, indicated localized stress redistribution prior to fracture. The external layers remained well-bonded with no visible delamination, confirming strong interlayer adhesion.

The triangular specimen (V2A/PLA_222) (Figure 7b) fractured along the cell junctions, following the principal stress paths inherent to its geometry. Sharp fracture edges and localized discoloration near cell vertices suggest stress accumulation leading to brittle failure. In contrast, the linear specimen (V3A/PLA_232) (Figure 7c) fractured predominantly along the printed line direction. The relatively smooth fracture surface and absence of delamination indicate that failure was governed by localized stress accumulation at the filament junctions, resulting in abrupt rupture.

**Figure 8:**
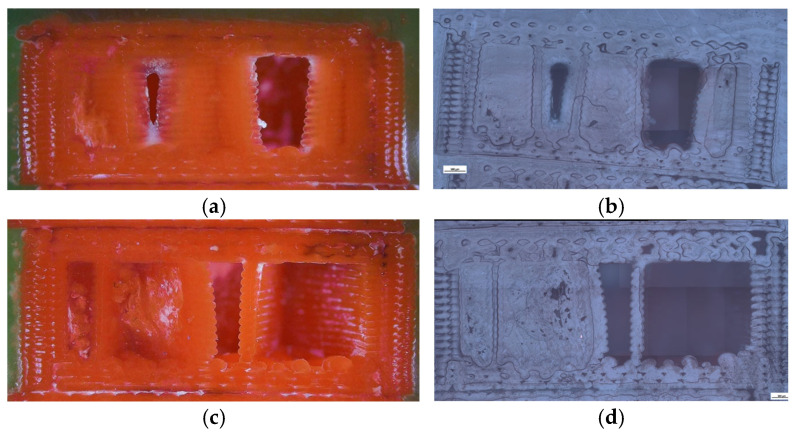

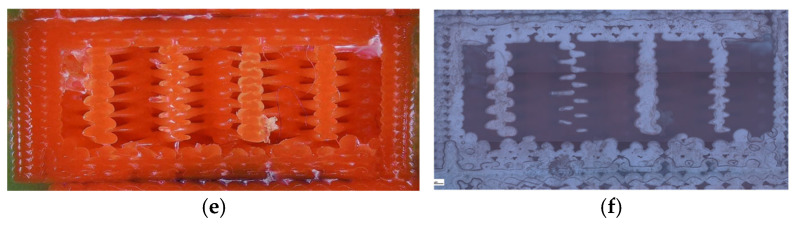
Optical micrographs of unfractured PLA specimens showing internal infill morphology: (**a**,**b**) hexagonal (V1B), (**c**,**d**) triangular (V2B), and (**e**,**f**) linear (V3B) structures. The hexagonal pattern shows minimal voids and good interlayer adhesion, whereas the triangular pattern exhibits larger pores near cell vertices.

To complement the mechanical findings, unfractured specimens were analyzed using a micro-camera and optical microscopy (Figure 8) to assess the internal morphology, porosity distribution, and interlayer adhesion quality.

Clear geometry-dependent differences were observed. The hexagonal sample (V1B) contained small voids aligned with the cell geometry, exhibiting stable sidewalls with only minor delamination near the upper layers. The triangular sample (V2B) displayed larger voids concentrated at the vertices, whereas the linear infill (V3B) showed a uniform structure with fewer, smaller voids mainly located near the model edges.

Figure 8 provides a qualitative comparison of pore distribution based on two-dimensional microstructural observations. While it does not allow direct quantification of volumetric porosity, it clearly illustrates relative differences in void presence between the investigated samples.

**Figure 9:**
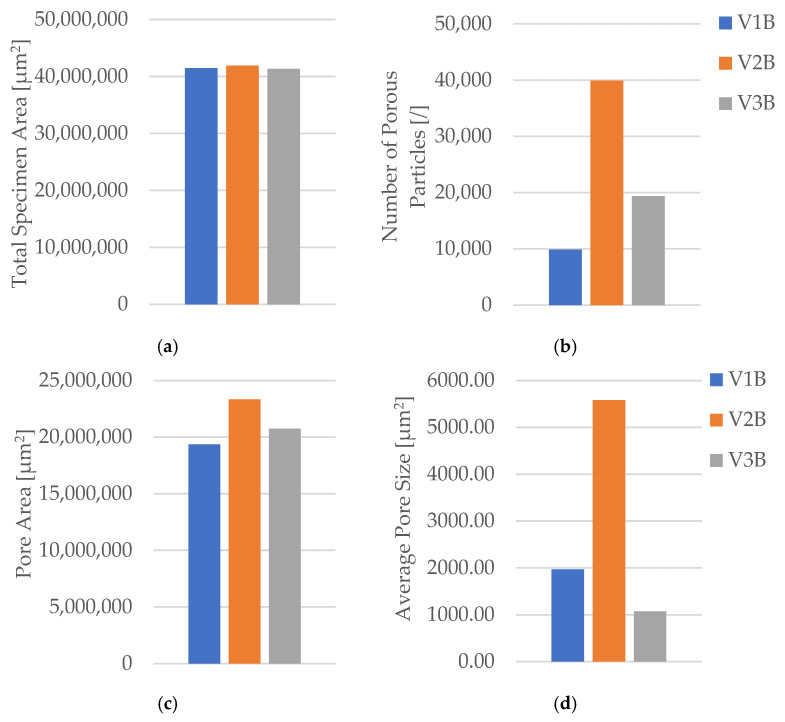
Quantitative microstructural analysis of PLA specimens with different infill geometries obtained using Fiji software: (**a**) total specimen area, (**b**) number of porous particles, (**c**) pore area, and (**d**) average pore size.

Quantitative microstructural data obtained using Fiji software ((ImageJ distribution, Version 2.14.0; LOCI, University of Wisconsin–Madison, WI, USA) (Table 4 and Figure 9) demonstrated a clear correlation between infill geometry and porosity.

The triangular pattern (V2B) exhibited the highest total pore count and pore area, resulting in the largest average pore size (5578.05 µm^2^) and the highest pore area fraction (55.64%). In contrast, the hexagonal structure (V1B) showed fewer and smaller voids, while the linear pattern (V3B) exhibited the lowest overall porosity.

The quantitative microstructural analysis supports the observed mechanical trends. The linear infill (V3A/PLA_232) exhibited the lowest porosity and the most uniform interlayer bonding, which corresponded to its highest tensile strength. Conversely, the triangular structure (V2A/PLA_222), characterized by the largest average pore size and highest pore area fraction, showed the lowest strength and stiffness, indicating that void formation critically impairs stress transfer across adjacent filaments. The hexagonal pattern (V1A/PLA_211) provided an intermediate behavior, where moderate porosity allowed for limited plastic deformation and higher ductility. These results confirm that mechanical performance in FDM-printed PLA is governed by a direct interplay between infill geometry, porosity distribution, and interlayer adhesion.

In summary, these findings highlight the critical influence of infill geometry on the mechanical and microstructural behavior of FDM-printed PLA. Optimizing internal architecture enables effective control over strength, stiffness, and ductility, providing a valuable framework for the design of lightweight, high-performance polymeric components. Such insights are particularly relevant for functional and load-bearing applications in mechanical, aerospace, and biomedical engineering. The correlation between lower porosity and improved tensile performance confirms that enhanced filament fusion and reduced void formation facilitate more efficient stress transfer across layers.

These results form the basis for further correlation analysis between pore morphology and apparent tensile properties, as discussed in Section 4.

#### 3.1.2. Influence of Infill Density on the Mechanical and Microstructural Behavior of 3D-Printed PLA Specimens

The infill density is one of the most influential parameters determining the mechanical strength, stiffness, and microstructural quality of FDM-printed polymer components. Variations in infill density affect the material continuity, void fraction, and efficiency of stress transfer between adjacent filaments. To evaluate this influence, three groups of PLA specimens with identical geometry (linear infill pattern, V3 series) but different infill densities—30% (V3A), 60% (V4A), and 100% (V5A)—were fabricated under identical printing conditions.

**Figure 10:**
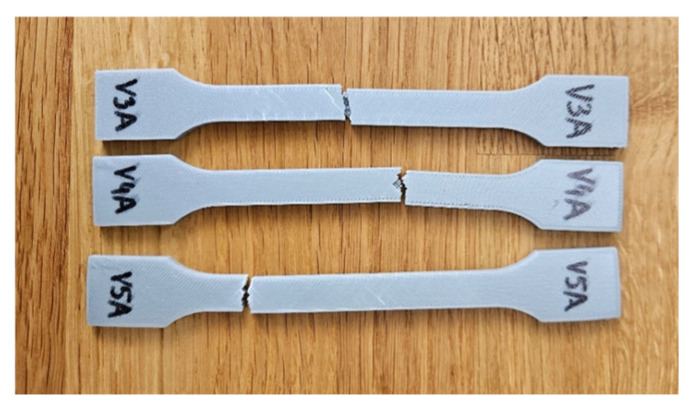
PLA tensile specimens printed with different infill densities: (V3A) 30%, (V4A) 60%, and (V5A) 100%. Increasing infill density reduces the internal void fraction and produces a more compact structure.

Figure 10 shows the appearance of the specimens after tensile testing, revealing clear differences in fracture morphology between low- and high-density structures.

**Figure 11:**
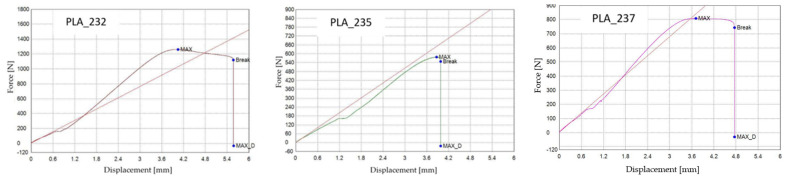
3D-printed PLA specimens tested: the force–displacement curves. The specimens PLA_232, PLA_2235, and PLA_237 are representative samples of case V3A, V4A, and V5A, respectively.

**Figure 12:**
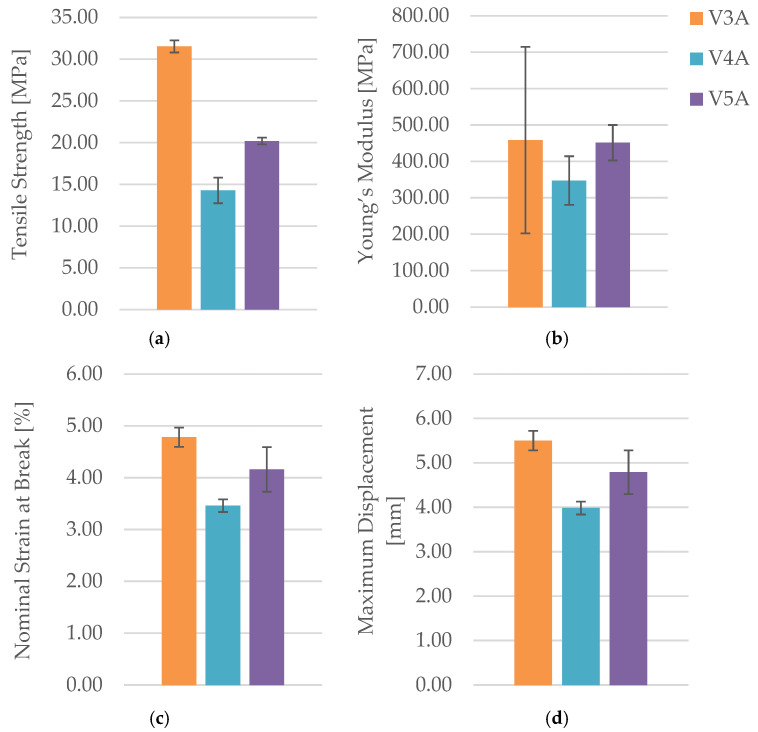
Comparison of tensile properties of PLA specimens with different infill densities: (**a**) tensile strength, (**b**) Young’s modulus, (**c**) nominal strain at break, and (**d**) maximum displacement. The 30% infill specimen (V3A/PLA_232) achieved the highest tensile strength, while the 60% infill (V4A/PLA_235) exhibited the weakest mechanical response.

The corresponding mechanical parameters obtained from the tensile tests are summarized in Table 5, while Figure 11 and Figure 12 compares the key mechanical indicators—tensile strength (σ), Young’s modulus (E), nominal strain at break (εₑ), and maximum displacement (Δl).

Figure 11 presents representative force–displacement (F−Δl) curves of FDM 3D-printed PLA specimens with a linear infill structure at different infill densities. The specimens PLA_232 (V3A, 30%), PLA_235 (V4A, 60%), and PLA_237 (V5A, 100%) are shown as representative samples for each case. The curves indicate that case V3A/PLA_232 exhibits the highest maximum force and the largest displacement at failure, which is consistent with the highest average tensile strength and strain at break reported in Table 5. In contrast, case V4A/PLA_235 shows a markedly lower load-bearing capacity and stiffness, while case V5A/PLA_237 achieves intermediate force and displacement values despite the fully dense infill. The differences in the curve profiles clearly demonstrate the influence of infill density on the mechanical response of the PLA specimens.

The dependence of tensile performance on infill density is non-monotonic. At 30% (V3A/PLA_232), favorable filament orientation and controlled porosity maximize strength; at 60% (V4A/PLA_235), partially filled cells create irregular load paths and stress localizations; at 100% (V5A/PLA_237), material continuity rises but interfacial constraints and residual stresses can promote premature cracking. The specimen with 30% infill (V3A/PLA_232) unexpectedly achieved the highest tensile strength (31.53 MPa) and stiffness (E = 458.76 MPa), combined with moderate ductility (εₑ = 4.78%, Δl = 5.50 mm). In contrast, the 60% infill specimen (V4A/PLA_235) exhibited the lowest mechanical response (σ = 14.28 MPa, E = 347.47 MPa, εₑ = 3.46%), likely due to stress localization within partially filled void regions and irregular interlayer bonding.

The fully dense specimen PLA_237 (V5A, 100%) demonstrated an intermediate tensile strength (20.20 MPa) and Young’s modulus (E = 451.46 MPa), suggesting that although material continuity was maximized, increased internal stress concentration at the fully compacted structure may have initiated premature fracture.

The observed maximum tensile strength at 30% linear infill is attributed to a combination of raster alignment with the loading direction and favorable void topology, which enables efficient axial load transfer and partial stress redistribution. At 60% infill, irregularly shaped void clusters and incomplete filament fusion act as stress concentrators, leading to premature failure. Although 100% infill provides higher material continuity, increased interlayer constraints and residual stresses can promote early crack initiation. It should be emphasized that tensile strength values for infill densities below 100% represent apparent strengths referenced to the gross cross-section.

**Figure 13:**
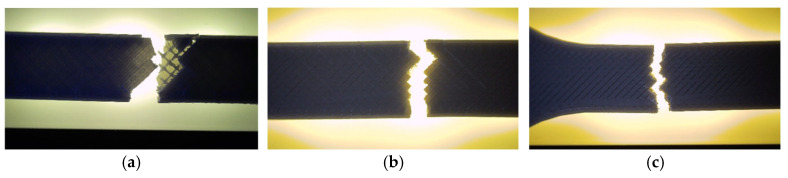
Fracture surfaces of PLA specimens after tensile testing: (**a**) 30% (V3A/PLA_232), (**b**) 60% (V4A/PLA_235), and (**c**) 100% (V5A/PLA_237). The intermediate-density specimen (V4A/PLA_235) shows brittle fracture features and poor interlayer bonding.

Fractographic analysis provided additional insight into the distinct failure mechanisms observed among the specimens (Figure 13). The 30% infill specimen (V3A/PLA_232) exhibited a relatively smooth fracture surface with limited delamination, suggesting effective load redistribution and stable filament fusion despite reduced material content. This behavior can be attributed to the stress redistribution effect associated with partially porous internal structures. At 30% infill, the presence of voids enables localized strain accommodation and delays the onset of brittle fracture, while excessive densification (100% infill) restricts internal stress relaxation and may promote microcrack initiation along the interlayer boundaries. The 60% infill specimen (V4A/PLA_235) revealed a brittle fracture with sharp edges and visible interlayer gaps, confirming incomplete filament bonding and localized stress accumulation at pore junctions. Conversely, the 100% infill specimen (V5A/PLA_237) demonstrated compact fracture morphology with strong interlayer adhesion but also exhibited microcracks originating from interfacial defects, indicating local overstressing within the highly compact structure.

Mechanistically, the 60% layout leaves ligaments and void clusters that act as micro-notches within the infill, concentrating stress at filament junctions. This explains the brittle-like features and the reduced tensile strength (σ) and Young’s modulus (E) despite higher material usage than 30%.

**Figure 14:**
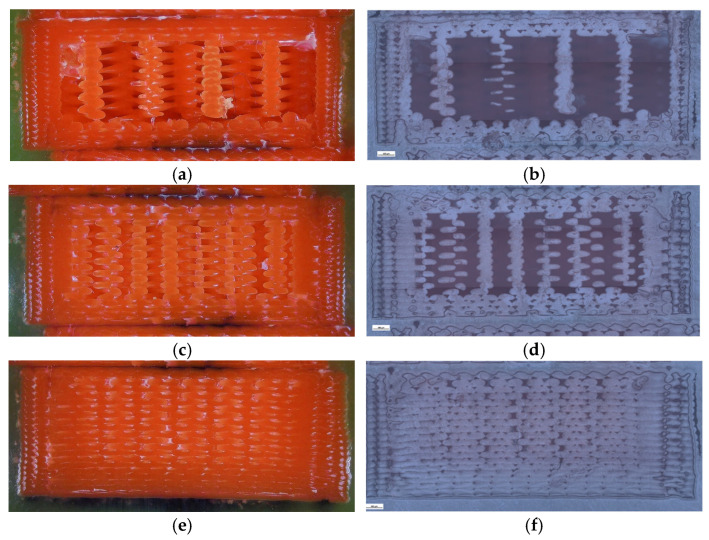
Optical micrographs of PLA specimens with different infill densities: (**a**,**b**) 30% (V3B), (**c**,**d**) 60% (V4B), and (**e**,**f**) 100% (V5B). The 100% infill specimen (V5B) exhibits the highest compactness and uniformity, while the 60% infill specimen (V4B) displays irregular void clusters.

To further support these findings, the unfractured specimens were examined using optical microscopy to assess the internal infill morphology, void distribution, and interlayer adhesion quality (Figure 14). Increasing the infill density visibly reduced the number and size of internal pores, while improving structural uniformity and contact between adjacent filaments. The 30% infill specimen (V3B) contained larger but evenly distributed pores, which contributed to partial stress dissipation during tensile loading. The 60% infill specimen (V4B) exhibited irregularly shaped void clusters that acted as stress concentrators, explaining its inferior mechanical response. The 100% infill specimen (V5B) displayed minimal visible porosity, although the dense filament packing may have induced microcrack initiation under localized stress accumulation.

**Figure 15:**
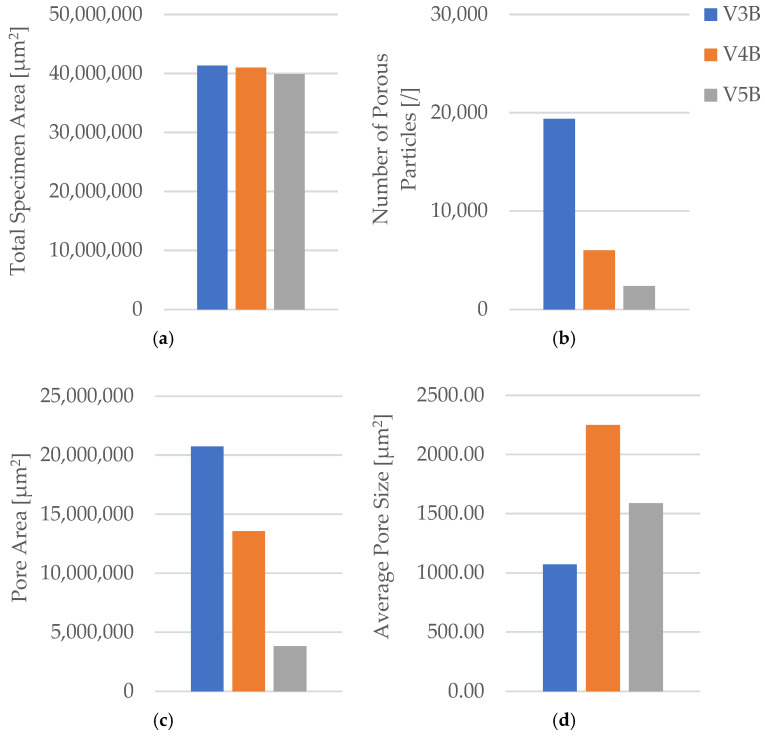
Quantitative microstructural analysis of PLA specimens with different infill densities obtained using Fiji software: (**a**) total specimen area, (**b**) number of porous particles, (**c**) pore area, and (**d**) average pore size.

Quantitative analysis of the microstructural features was performed using Fiji software (Table 6, Figure 15), confirming the relationship between density, pore distribution, and average pore size.

The pore count and total pore area decrease with increasing infill density, while the average pore size exhibits a non-monotonic trend, reflecting local variations in interlayer fusion and thermal history. This indicates that densification improves compactness overall, but micro-scale processing conditions still govern defect morphology.

The microstructural results are consistent with the mechanical trends. Specimens with higher infill density exhibited a more compact structure, stronger interlayer bonding, and fewer voids, while intermediate-density samples showed irregular pore clusters that weakened local adhesion. The non-monotonic mechanical behavior suggests that excessive densification can lead to stress concentration and early crack initiation, while lower density allows limited energy absorption due to larger, evenly distributed voids.

In summary, increasing infill density from 30% to 100% did not result in a simple linear improvement in tensile performance. Instead, the optimal mechanical response was achieved at 30% infill, where balanced filament orientation and moderate porosity contributed to superior strength and ductility. These findings confirm that mechanical behavior in FDM-printed PLA is controlled not only by infill density but also by the quality of interlayer fusion and pore morphology, consistent with the correlations reported by Guessasma et al. [62].

Note that tensile strength (σ) and Young’s modulus (E) are apparent values referenced to the gross cross-section; thus, comparisons are meaningful within the present geometry/density framework. The superior apparent response at 30% infill is attributed to raster alignment and void topology that enhance axial load transfer despite reduced solid fraction.

The observed non-linear behavior, particularly the lower tensile strength of PLA specimens with 60% infill compared to those with 30% and 100%, can be attributed to microstructural inhomogeneity and local residual stresses formed during the deposition process. Microscopic examination confirmed that in the 60% infill specimens, irregular interlayer bonding and localized stress concentrations occurred, which likely promoted premature fracture. Therefore, this deviation was interpreted as a process-related anomaly, rather than an intrinsic material characteristic.

#### 3.1.3. Influence of Exposure to Mineral Engine Oil on the Mechanical and Microstructural Behavior of 3D-Printed PLA Specimens

The environmental exposure of FDM-printed polymer components to chemical agents such as mineral oils can significantly alter their mechanical integrity and microstructural morphology. To evaluate this influence, tensile tests were conducted on PLA specimens with hexagonal infill geometry (30% density) before and after 7 days of immersion in mineral motor oil. Two specimen groups were analyzed: V1A (unexposed) and V6A (7 days oil-exposed).

**Figure 16:**
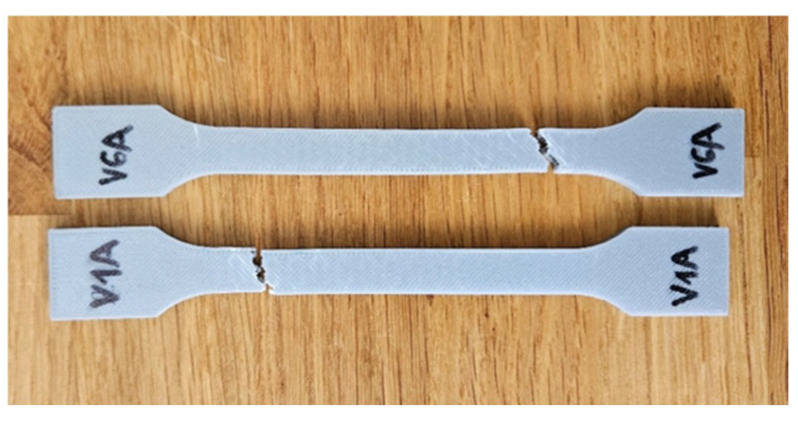
PLA tensile specimens printed with hexagonal infill (30% density) after tensile testing: (V1A/PLA_211) unexposed sample, (V6A/PLA_214) sample after 7 days of exposure to mineral motor oil.

**Figure 17:**
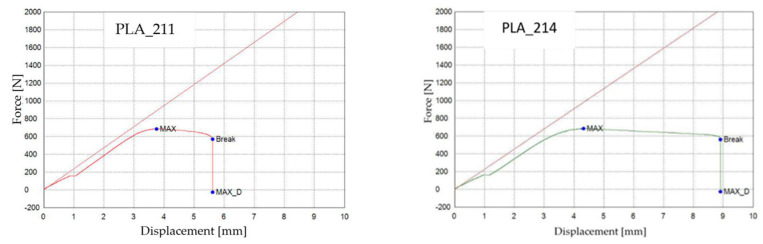
3D-printed PLA specimens tested: the force–displacement curves. The specimens PLA_211 and PLA_214 are representative samples of case V1A and V6A, respectively.

**Figure 18:**
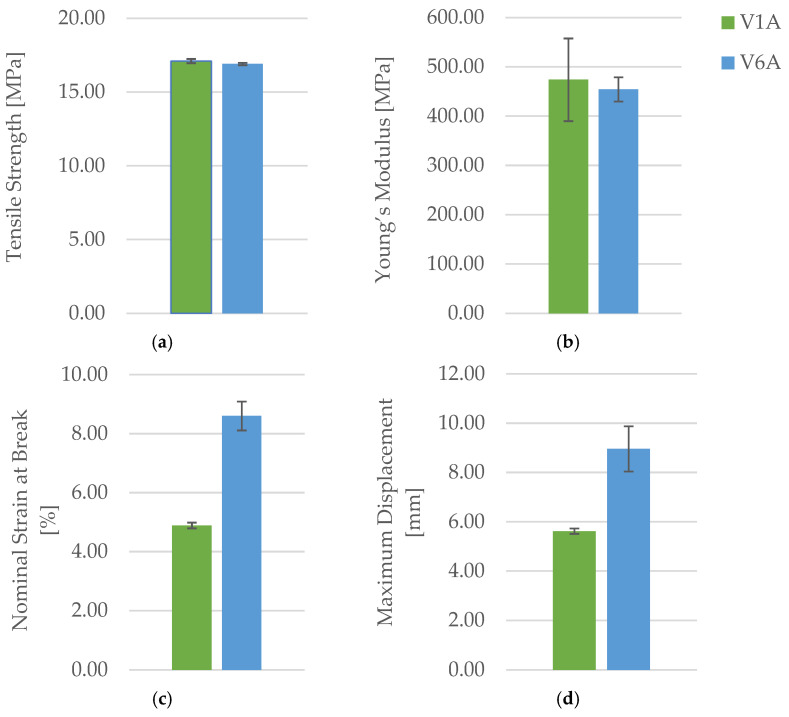
Comparison of tensile properties of PLA specimens before and after 7-day exposure to mineral motor oil: (**a**) tensile strength, (**b**) Young’s modulus, (**c**) nominal strain at break, and (**d**) maximum displacement. Oil exposure increased ductility while slightly reducing stiffness and tensile strength.

Figure 16 presents the PLA specimens after tensile testing, showing clear visual differences in the fracture morphology between the unexposed and oil-exposed samples. The corresponding mechanical parameters are summarized in Table 7, while the comparative analysis of the key mechanical indicators—maximum force (F), tensile strength (σ), Young’s modulus (E), nominal strain at break (εₑ), and maximum displacement (Δl)—is illustrated in Figure 17 and Figure 18.

Figure 17 shows the representative force–displacement (F−Δl) curves of FDM 3D-printed PLA specimens with a hexagonal infill structure at 30% infill density, before and after exposure to mineral oil. The specimens PLA_211 (V1A, unexposed) and PLA_214 (V6A, 7 days exposed) are presented as representative samples of each condition. The curves indicate that mineral oil exposure does not significantly affect the maximum force or tensile strength, which remain comparable for both cases, in agreement with the average values reported in Table 7. However, the exposed specimen (V6A) exhibits a noticeably higher displacement and strain at break, indicating increased ductility after exposure. These changes are reflected in the altered slope and extended deformation region of the force–displacement curve, demonstrating the influence of mineral oil exposure on the mechanical response of the PLA specimens.

The results summarized in Table 7 and illustrated in Figure 17 and Figure 18 indicate that exposure to mineral oil slightly affected the mechanical behavior of the PLA specimens.

The tensile strength decreased marginally from 17.10 MPa (V1A/PLA_211) to 16.90 MPa (V6A/PLA_214), while the Young’s modulus dropped from 473.71 MPa to 454.13 MPa, indicating a small but measurable reduction in stiffness. Conversely, the nominal strain at break increased significantly from 4.89% to 8.60%, and the maximum displacement rose from 5.62 mm to 8.96 mm, confirming a pronounced improvement in ductility and elongation capacity.

These findings suggest that mineral oil acts as a mild plasticizing medium: absorbed molecules penetrate between polymer chains, reducing intermolecular cohesion and enhancing chain mobility. Consequently, the material exhibited slightly lower stiffness and strength but improved flexibility and energy absorption capability.

**Figure 19:**
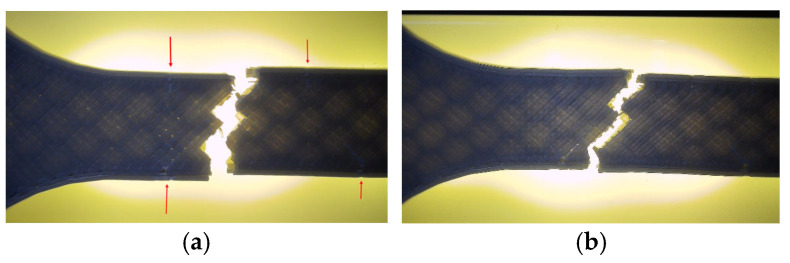
Fracture surfaces of PLA specimens after tensile testing: (**a**) V1A—unexposed (PLA_211), brittle fracture with clean separation; (**b**) V6A—after 7 days in mineral oil (PLA_214), showing ductile failure and localized plastic deformation.

Fractographic examination revealed distinct differences in the failure mechanisms between the unexposed and oil-exposed specimens (Figure 19).

The unexposed specimen PLA_211 (V1A) fractured at the narrowest cross-section, exhibiting a clean and predominantly brittle fracture with minor tearing along the inner cell walls. Local color variations at the hexagonal junctions indicated stress redistribution prior to failure, while the outer surfaces remained well bonded, confirming strong interlayer adhesion.

In contrast, the oil-exposed specimen PLA_214 (V6A) displayed a rougher fracture surface with visible shear zones, indicating enhanced plastic deformation. The crack propagation followed weakened filament junctions within the hexagonal structure, where localized softening and partial delamination were observed.

**Figure 20:**
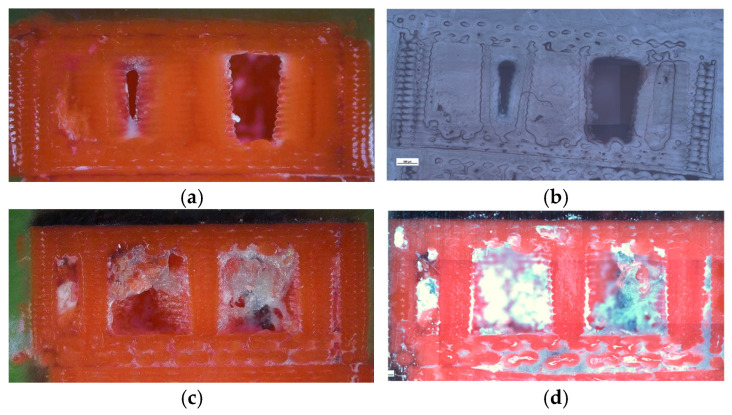
Optical micrographs of unfractured PLA specimens with hexagonal infill (30%): (**a**,**b**) unexposed (V1B); (**c**,**d**) after 7 days of mineral oil exposure (V6B). Oil absorption caused pore filling and slight delamination between layers.

Optical microscopy was used to examine unfractured specimens and assess morphological changes induced by oil exposure (Figure 20).

The unexposed specimen (V1B) showed small, regularly distributed voids corresponding to the hexagonal infill structure and well-formed interlayer bonding. Only minor delamination was observed near upper layers.

**Figure 21:**
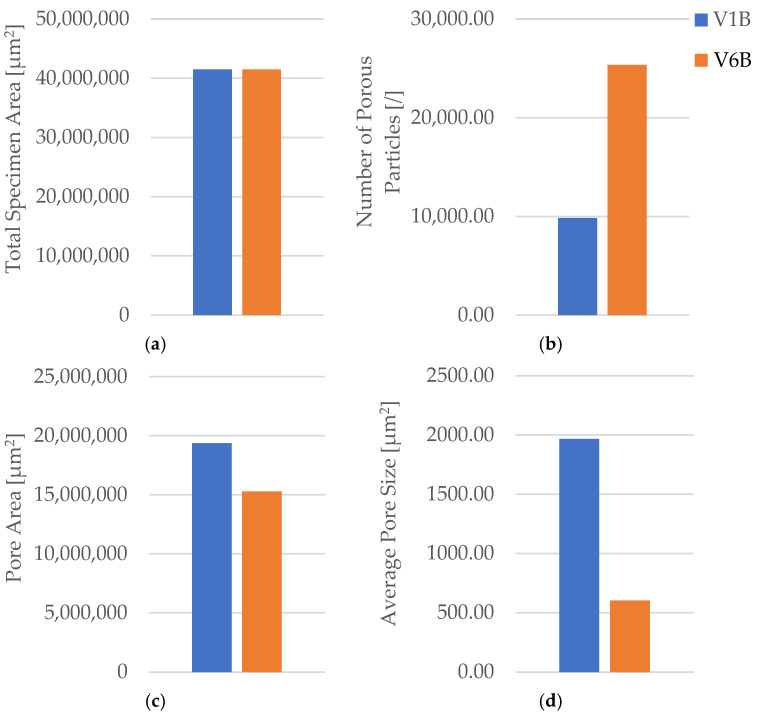
Quantitative microstructural analysis of PLA specimens before and after mineral oil exposure: (**a**) total specimen area, (**b**) number of porous particles, (**c**) pore area, and (**d**) average pore size. Oil exposure increased pore count but reduced pore size and total pore area, leading to a finer and more uniform void distribution.

The oil-exposed specimen (V6B) exhibited partially oil-filled pores and mild interlayer separation, particularly near the lower specimen region. These observations confirm that mineral oil infiltrated the voids, weakened interlayer adhesion, and induced local softening, resulting in greater ductility but reduced rigidity. Quantitative image analysis using Fiji software (Table 8, Figure 21) confirmed significant microstructural changes after oil exposure.

The total specimen area remained nearly constant, while the number of pores increased from 9841 (V1B) to 25, 338 (V6B)—a 2.6-fold rise. Despite the higher pore count, the total pore area decreased from 19.36 × 10^6^ µm^2^ to 15.27 × 10^6^ µm^2^, and the average pore size reduced from 1967.24 µm^2^ to 602.74 µm^2^, indicating the formation of finer and more uniformly distributed voids within the matrix.

The combined mechanical and microstructural results demonstrate that exposure to mineral motor oil induces partial softening and microstructural refinement of the PLA matrix. A minor decrease in tensile strength (−1.2%) and Young’s modulus (−4.1%) was accompanied by a substantial increase in elongation (+76%), reflecting plasticization of the polymer network.

The correlation between the increased ductility and refined microstructure indicates that oil absorption reorganized the internal structure, facilitating stress relaxation and energy dissipation during loading.

Although PLA is biodegradable under specific environmental conditions, the short-term oil exposure applied in this study did not induce biodegradation-related mass loss or surface degradation; the observed changes are attributed to physical plasticization rather than chemical or biological degradation.

These findings are consistent with the softening mechanisms reported by Pascual-González et al. [61] and Guessasma et al. [62], where exposure to liquid media enhanced polymer flexibility by reducing secondary intermolecular interactions.

Compared to the results in Section 3.1.1 and Section 3.1.2, where infill geometry and density primarily affected the mechanical response through structural continuity and void fraction, the present results show that environmental exposure influences the PLA matrix itself, introducing molecular-level softening rather than geometric weakening.

In summary, immersion in mineral motor oil slightly reduces stiffness and strength but significantly enhances ductility, confirming that chemical–environmental exposure can substantially modify the mechanical behavior of FDM-printed PLA through matrix plasticization and microstructural adaptation.

These results highlight the importance of considering lubricant compatibility when designing PLA-based components intended for service in oil-contact or hydrocarbon-rich environments.

Tensile behavior of FDM-PLA is governed by two coupled levers: (i) structural topology (raster orientation, cell junction sharpness, void topology) and (ii) matrix conditioning (oil-induced plasticization). Aligning the raster with the load and avoiding sharp junctions improves σ/E, while judicious density selection prevents stress localization (the 30% linear case outperforms 60–100% in σ). Oil exposure introduces molecular-level softening and defect refinement (the pore count increased by approximately 2.6 times, while the pore size decreased by approximately 3.3 times), trading small losses in σ/E for a ~76% gain in ductility.

Limitations. The present results are specific to the selected PLA grade and printing parameters. Potential confounders include environmental humidity, filament moisture content, and build-plate temperature history. Oil exposure was limited to 7 days; longer conditioning or different lubricants (e.g., synthetic or bio-based oils) may alter the magnitude of plasticization. These aspects warrant future study.

In addition, the use of apparent stress and modulus for <100% infill implies geometry-dependent values that are not directly comparable to fully dense, isotropic datasheet properties. Future work should complement apparent metrics with normalization to effective load-bearing area and with larger sample sizes to increase statistical power.

### 3.2. Mechanical Parameters and Microstructural Response of 3D-Printed PLA+CF Specimens

The mechanical behavior and microstructural response of FDM 3D-printed PLA+CF specimens were systematically evaluated by examining the influence of infill structure geometry (Section 3.2.1), the infill density of the linear pattern (Section 3.2.2), and the duration of exposure to mineral motor oil in specimens with hexagonal infill structures (Section 3.2.3).

Across Section 3.2.1, Section 3.2.2 and Section 3.2.3, specimen codes are used as follows: geometry series (30% infill): V7A/V7B = hexagonal, V8A/V8B = triangular, V9A/V9B = linear. Density series (linear infill): V9A–V11A (tensile) and V9B–V11B (microstructure) correspond to 30%, 60%, and 100%. Oil exposure (hexagonal, 30%): V7A/V7B = unexposed; V12A/V12B = 7-day exposure. Suffix A = tensile specimens; B = unfractured for microscopy.

#### 3.2.1. Influence of Infill Geometry on the Mechanical and Microstructural Behavior of 3D-Printed PLA+CF Specimens

The geometry of the internal infill structure plays a decisive role in determining the load-bearing capacity, stiffness, and fracture behavior of FDM-printed composite components. For PLA reinforced with short carbon fibers (PLA+CF), the effect of infill geometry is further amplified by the partial alignment of fibers along the extrusion direction, which governs both the mechanical response and the resulting microstructural morphology.

**Figure 22:**
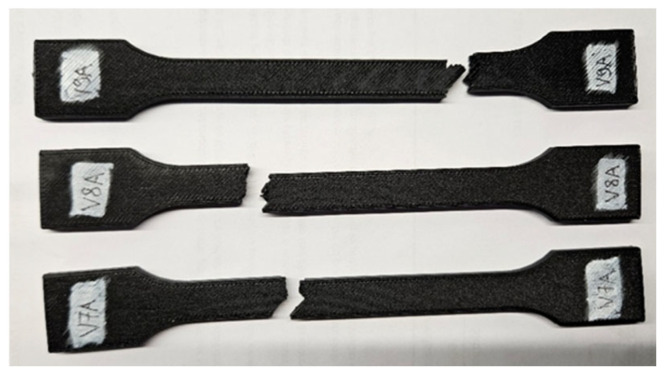
PLA+CF tensile specimens printed with different infill geometries: hexagonal (V7A/PLA+CF_513), triangular (V8A/PLA+CF_522), and linear (V9A/ PLA+CF_532), all with 30% infill density. The fracture surfaces reveal geometry-dependent differences in filament failure and fiber–matrix interaction.

**Figure 23:**
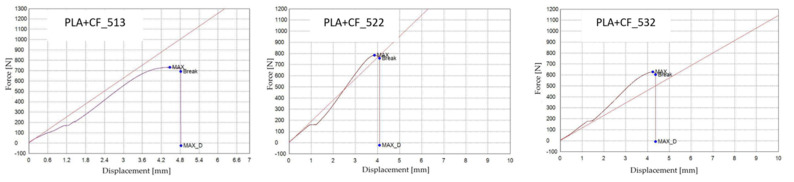
3D-printed PLA+CF specimens tested: the force–displacement curves. The specimens PLA+CF_513, PLA+CF_522, and PLA+CF_232 are representative samples of case V7A, V8A, and V9A, respectively.

**Figure 24:**
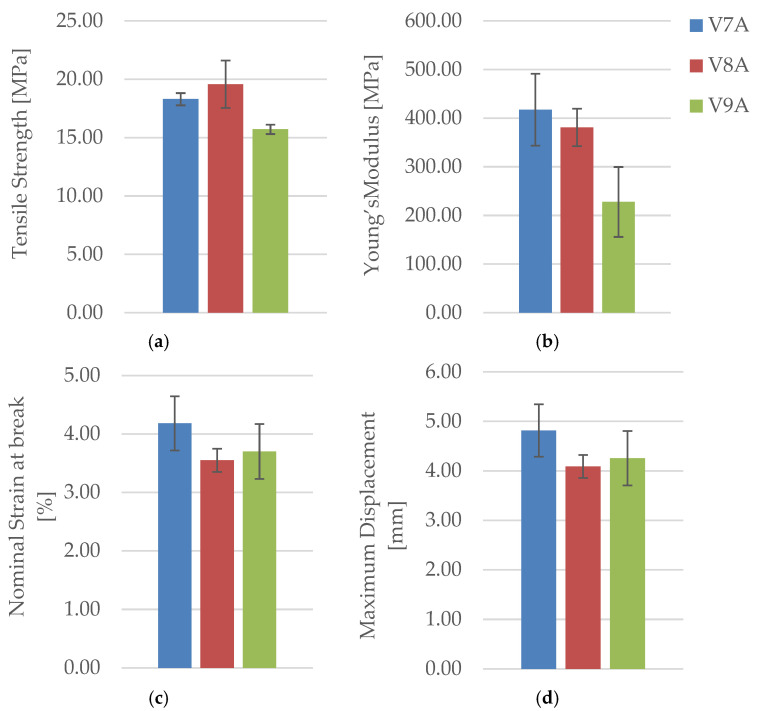
Comparison of tensile properties of PLA+CF specimens with different infill geometries: (**a**) tensile strength, (**b**) Young’s modulus, (**c**) nominal strain at break, and (**d**) maximum displacement. The triangular pattern (V8A) exhibits the highest tensile strength, while the hexagonal pattern (V7A) demonstrates the best combination of stiffness and ductility.

Figure 22 presents the PLA+CF specimens after tensile testing, printed with three distinct infill geometries: hexagonal (V7A/PLA+CF_513), triangular (V8A/PLA+CF_522), and linear (V9A/PLA+CF_532). The corresponding tensile test results are summarized in Table 9, and the comparative analysis of the principal mechanical parameters—maximum force (F), tensile strength (σ), Young’s modulus (E), nominal strain at break (εₑ), and maximum displacement (Δl)—is shown in Figure 23 and Figure 24.

The obtained results clearly demonstrate that the infill geometry exerts a significant influence on the tensile performance of PLA+CF specimens. The triangular infill (V8A/PLA+CF_522) exhibited the highest tensile strength (19.58 MPa), attributed to the efficient load transfer through short, stiff cell junctions that minimize stress concentration. The hexagonal structure (V7A/PLA+CF_513) showed slightly lower strength (18.30 MPa) but the highest ductility (εₑ = 4.82%), confirming its ability to absorb deformation energy and delay catastrophic failure. The linear infill (V9A/PLA+CF_532) displayed the lowest mechanical performance (σ = 15.72 MPa, E = 228.84 MPa), likely due to stress accumulation along filament boundaries and weaker interlayer bonding.

The addition of short carbon fibers enhanced the stiffness and dimensional stability compared to pure PLA (Section 3.1.1), although the magnitude of improvement depended strongly on the geometric arrangement of the internal structure. The triangular and hexagonal patterns provided better stress redistribution and interlayer fusion, whereas the linear pattern—although aligned with the loading axis—showed premature interfacial separation and lower stress tolerance.

The observed differences were further interpreted through a combined mechanical–microstructural correlation analysis.

Figure 23 shows representative force–displacement (F−Δl) curves of FDM 3D-printed PLA+CF specimens with different infill geometries at 30% infill density. The specimens PLA+CF_513 (V7A, hexagonal), PLA+CF_522 (V8A, triangular), and PLA+CF_531 (V9A, linear) are presented as representative samples. The curves indicate that the hexagonal infill exhibits the highest load-bearing capacity and displacement at failure, whereas the triangular and linear infills show lower force levels and reduced deformation. These differences confirm the influence of infill geometry on the mechanical response of PLA+CF specimens.

**Figure 25:**
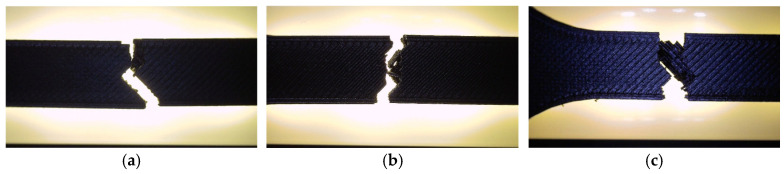
Fracture surfaces of PLA+CF specimens after tensile testing: (**a**) V7A—hexagonal structure; (**b**) V8A—triangular structure; (**c**) V9A—linear structure. The fracture morphology reflects the interplay between infill topology and fiber–matrix adhesion.

Fractographic analysis (Figure 25) revealed distinct failure mechanisms for each geometry. The hexagonal specimen (V7A/PLA+CF_513) fractured through a combination of matrix cracking and fiber pull-out, with smooth fracture regions surrounded by localized shear zones. The triangular specimen (V8A/PLA+CF_522) exhibited brittle-like fracture features at the cell vertices, with visible fiber imprints and microvoids at interlayer junctions. The linear specimen (V9A/PLA+CF_532) showed a more uniform fracture surface dominated by filament splitting and limited fiber bridging, indicating interfacial decohesion between fibers and the PLA matrix.

**Figure 26:**
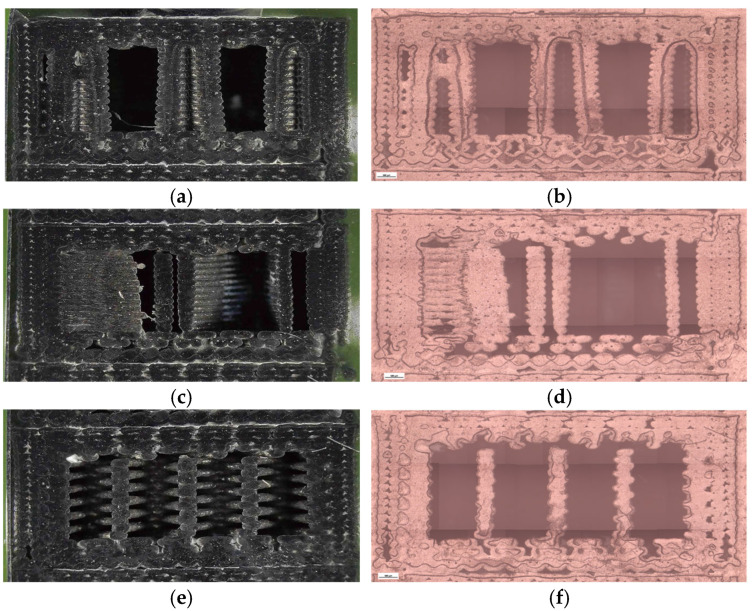
Optical micrographs of unfractured PLA+CF specimens showing internal infill morphology: (**a**,**b**) hexagonal (V7B), (**c**,**d**) triangular (V8B), and (**e**,**f**) linear (V9B) structures. The triangular infill exhibits the most compact structure with reduced void fraction.

To complement the mechanical results, unfractured specimens were analyzed using optical microscopy to assess internal morphology, porosity distribution, and fiber–matrix interfacial bonding. Unfractured specimens were further examined using optical microscopy (Figure 26) to assess the internal porosity and fiber distribution. The hexagonal structure (V7B) exhibited moderately distributed voids and well-bonded interlayers. The triangular infill (V8B) showed compact regions with minimal porosity, while the linear infill (V9B) revealed elongated voids parallel to the filament orientation, indicating incomplete fusion between adjacent rasters.

The triangular infill (V8B) showed the smallest and most uniformly distributed voids, which correspond to improved filament coalescence and reduced fiber–matrix debonding zones.

**Figure 27:**
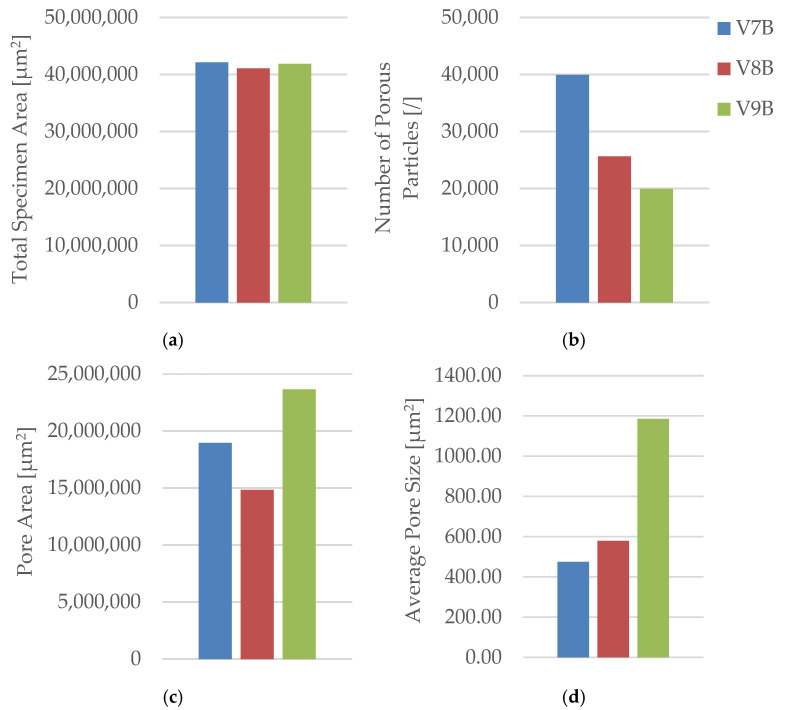
Quantitative microstructural analysis of PLA+CF specimens with different infill geometries obtained using Fiji software: (**a**) total specimen area, (**b**) number of porous particles, (**c**) pore area, and (**d**) average pore size.

Quantitative image analysis using Fiji software provided detailed pore metrics, confirming geometry-dependent variations in porosity and void morphology (Table 10, Figure 27).

Quantitative microstructural data obtained using Fiji software (Table 10, Figure 27) demonstrated a direct correlation between infill geometry and porosity distribution. The quantitative analysis confirms that the triangular infill (V8B) exhibited the lowest porosity and smallest average pore size, correlating with its highest tensile strength and stiffness. The linear infill (V9B) presented the largest pore area fraction and highest average pore size, explaining its reduced mechanical performance. The hexagonal structure (V7B) provided an intermediate balance between porosity and ductility, indicating that moderate internal voids can enhance energy absorption without severely compromising strength.

In summary, the results demonstrate that infill geometry substantially affects both the mechanical performance and microstructural integrity of PLA+CF composites. The triangular pattern yielded the highest tensile strength due to superior compaction and stress distribution, while the hexagonal pattern offered enhanced ductility and energy absorption capacity. The linear pattern was the least effective configuration, primarily due to its higher porosity and reduced interlayer fusion. These findings highlight the necessity of optimizing internal geometry in carbon-fiber-reinforced FDM structures to achieve a desired balance between stiffness, strength, and ductility. The correlation between reduced porosity and enhanced tensile performance confirms that improved interlayer fusion, controlled fiber dispersion, and optimized filament alignment jointly determine the superior mechanical integrity of PLA+CF composites.

Compared to pure PLA (Section 3.1.1), the addition of short carbon fibers enhanced overall stiffness and dimensional stability, although the relative influence of infill geometry on ductility became more pronounced due to the reduced polymer chain mobility within the reinforced matrix.

#### 3.2.2. Influence of Infill Density on the Mechanical and Microstructural Behavior of 3D-Printed PLA+CF Specimens

The infill density is a critical factor determining the mechanical strength, stiffness, and internal integrity of FDM-printed composite parts. In the case of PLA reinforced with short carbon fibers (PLA+CF), changes in infill density not only modify the effective load-bearing area but also affect the degree of filament consolidation, porosity distribution, and fiber orientation within the printed matrix. These factors collectively influence stress transfer efficiency and fracture behavior under tensile loading.

**Figure 28:**
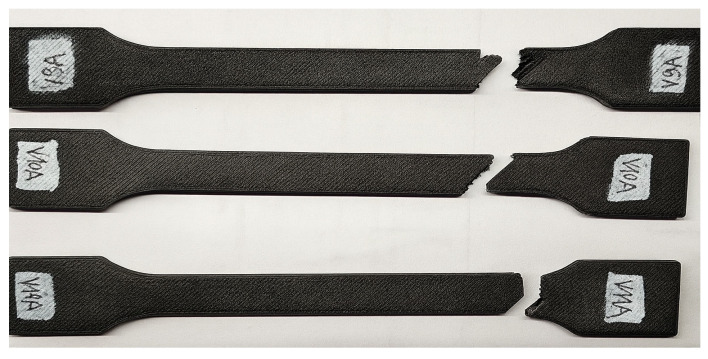
PLA+CF tensile specimens printed with different infill densities: (V9A) 30%, (V10A) 60%, and (V11A) 100%. The increase in infill density reduces void size and enhances filament packing compactness.

Three groups of PLA+CF specimens with identical geometry (linear infill pattern) but different infill densities—30% (V9A), 60% (V10A), and 100% (V11A)—were fabricated under identical process conditions. The specimens were printed in the same build orientation and raster direction to isolate the effect of density on mechanical and microstructural performance. Figure 28 shows the appearance of the specimens after tensile testing.

**Figure 29:**
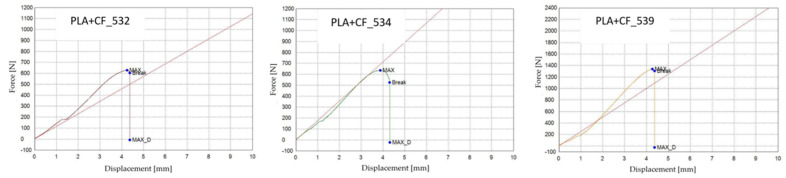
3D-printed PLA+CF specimens tested: the force–displacement curves. The specimens PLA+CF_532, PLA+CF_535, and PLA+CF_239 are representative samples of case V9A, V10A, and V11A, respectively.

**Figure 30:**
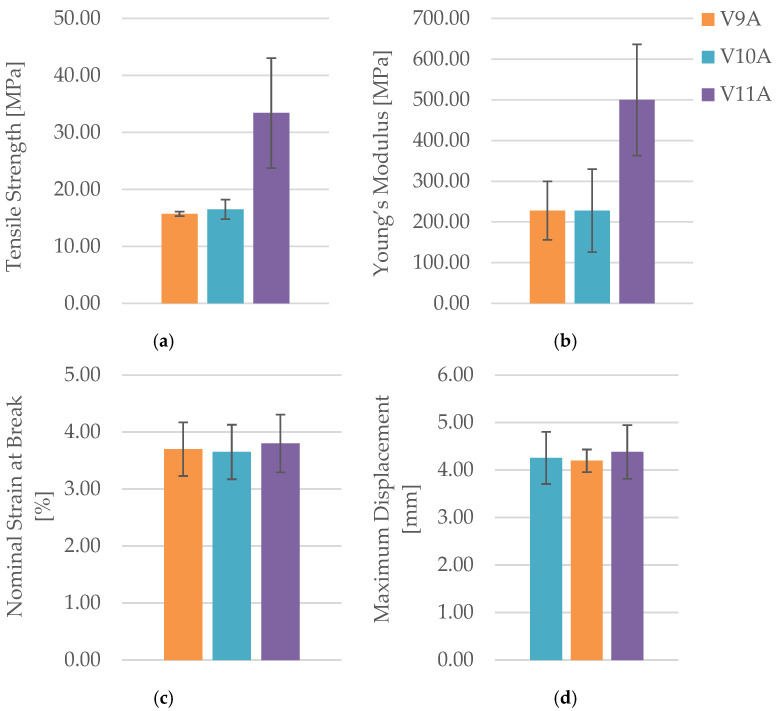
Comparison of tensile properties of PLA+CF specimens with different infill densities: (**a**) tensile strength, (**b**) Young’s modulus, (**c**) nominal strain at break, and (**d**) maximum displacement. The mechanical performance increases markedly with higher infill density, peaking at 100%.

The corresponding mechanical parameters obtained from the tensile tests are summarized in Table 11, while Figure 29 and Figure 30 compares the principal mechanical indicators—maximum force (F), tensile strength (σ), Young’s modulus (E), nominal strain at break (εₑ), and maximum displacement (Δl).

Figure 29 presents representative force–displacement (F−Δl) curves of FDM 3D-printed PLA+CF specimens with a linear infill pattern at different infill densities. The specimens PLA+CF_532 (V9A, 30%), PLA+CF_535 (V10A, 60%), and PLA+CF_537 (V11A, 100%) are shown as representative samples. The curves indicate a clear increase in maximum force with increasing infill density, with the fully dense specimens (V11A) exhibiting the highest load-bearing capacity. Differences in the deformation behavior further confirm the significant influence of infill density on the mechanical response of PLA+CF specimens.

The obtained results demonstrate a monotonic increase in tensile strength and stiffness with increasing infill density. The 100% infill specimen (V11A/PLA+CF_539) achieved the highest tensile strength (33.41 MPa) and Young’s modulus (499.89 MPa), approximately double those of the 30% infill specimen (V9A/PLA+CF_532). This improvement is attributed to the elimination of large internal voids and the formation of a continuous load path through the fiber-reinforced matrix. The intermediate-density specimen PLA+CF_535 (V10A, 60%) exhibited moderate mechanical performance (σ = 16.51 MPa, E = 228.13 MPa), indicating partial improvement in interlayer bonding but still containing irregular pores acting as local stress concentrators.

Figure 30b clearly illustrates the sharp rise in stiffness from 60% to 100% infill, consistent with the near doubling of the modulus in Table 11.

Despite the higher material content, the nominal strain at break remained in a narrow range (3.6–3.8%), reflecting the intrinsic stiffness of the carbon-fiber-reinforced PLA matrix. The modest increase in elongation for the 100% specimen (Δl = 4.38 mm) suggests that enhanced interlayer cohesion allows limited plastic deformation prior to fracture, even in the dense composite structure.

**Figure 31:**
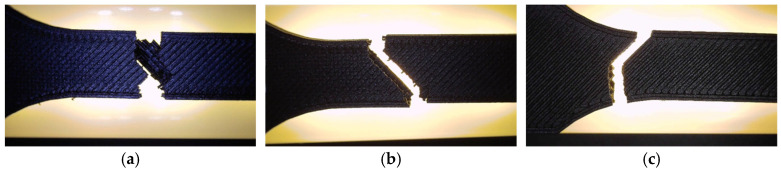
Fracture surfaces of PLA+CF specimens after tensile testing: (**a**) V9A—30%, (**b**) V10A—60%, (**c**) V11A—100% infill density. Increasing infill density enhances layer cohesion and reduces interfacial cracking.

Fractographic analysis (Figure 31) provided further insight into the failure mechanisms as a function of infill density.

The 30% infill specimen (V9A) exhibited a rough fracture surface with extensive void regions and partially pulled-out fibers, indicating interlayer decohesion. The 60% specimen PLA+CF_535 (V10A) displayed a mixed fracture morphology characterized by partially fused filaments and microvoid coalescence at fiber-rich zones. In contrast, the 100% infill specimen PLA+CF_539 (V11A) showed a compact fracture surface with well-bonded layers, clear fiber pull-out traces, and localized shear bands—features typical of ductile–brittle transitions in stiffened polymer composites.

**Figure 32:**
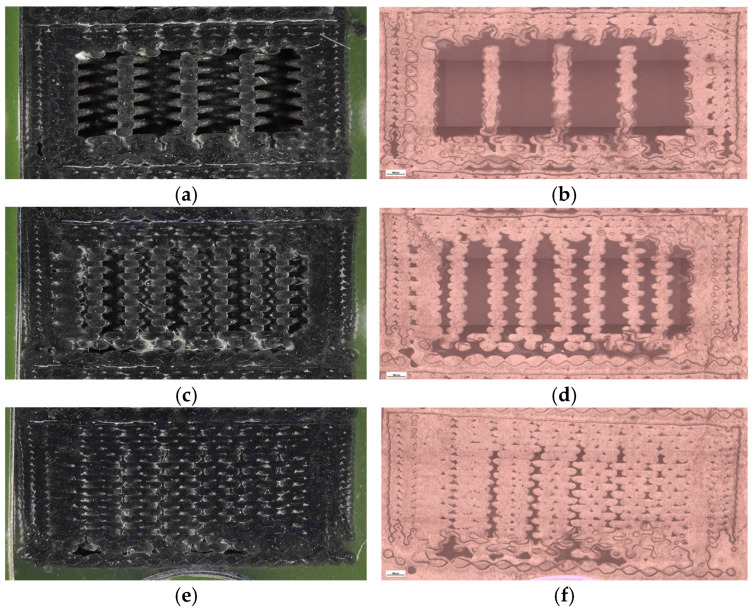
Optical micrographs of PLA+CF specimens with different infill densities showing internal morphology: (**a**,**b**) 30% (V9B), (**c**,**d**) 60% (V10B), and (**e**,**f**) 100% (V11B). Increasing infill density improves structural continuity and interlayer bonding, while reducing void content.

The visible reduction in interlayer gaps from (a) to (c) correlates with the improved bonding observed in optical microscopy (Figure 32). To complement the fractographic analysis, unfractured specimens were observed under optical microscopy to evaluate internal structure uniformity and pore distribution.

The 30% infill specimen (V9B) contained elongated voids oriented along the filament direction, suggesting incomplete fusion. The 60% specimen (V10B) exhibited irregularly shaped pores clustered near the midplane. The 100% specimen (V11B) displayed minimal porosity, compact raster alignment, and uniformly distributed short fibers embedded within the PLA matrix.

**Figure 33:**
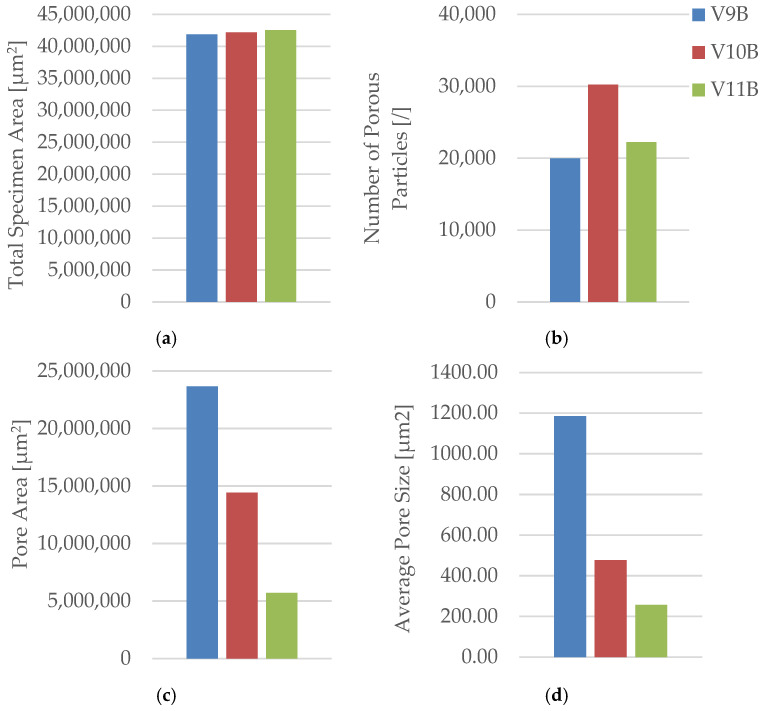
Quantitative microstructural analysis of PLA+CF specimens with different infill densities obtained using Fiji software: (**a**) total specimen area, (**b**) number of porous particles, (**c**) pore area, and (**d**) average pore size.

Quantitative image analysis using Fiji software (Table 12 and Figure 33) confirmed a strong inverse correlation between infill density and total pore area.

The pore count increased slightly from 19,955 in V9B (30%) to 30,242 in V10B (60%) due to irregular partial fusion, and then decreased to 22,235 in V11B (100%), where nearly complete interlayer bonding was achieved.

Despite this non-monotonic variation in pore count, the total pore area was reduced by more than 75% between 30% and 100% infill, confirming that higher density leads to a more compact and mechanically stable internal structure.

The steep reduction in total pore area at 100% infill indicates nearly complete material consolidation.

Compared to pure PLA (Section 3.1.2), the PLA+CF composite exhibited a more linear and predictable strengthening trend with density. This can be attributed to the stabilizing effect of carbon fibers, which reinforce the interlayer junctions and mitigate the internal stresses that can otherwise lead to premature crack initiation in unreinforced polymers.

In summary, the results demonstrate that increasing the infill density in PLA+CF specimens significantly enhances tensile strength and stiffness while reducing internal porosity. The best overall mechanical performance was achieved at 100% infill, where improved filament coalescence, reduced void fraction, and uniform fiber distribution resulted in the highest apparent modulus and tensile strength. The correlation between the densification of the internal structure and the improved mechanical response confirms that optimizing the infill density is essential for achieving high-performance FDM-printed composites.

These results are of particular importance for engineering applications where high stiffness and dimensional stability are required, such as in functional or load-bearing FDM components.

#### 3.2.3. Influence of Exposure to Mineral Engine Oil on the Mechanical and Microstructural Behavior of 3D-Printed PLA+CF Specimens

The environmental exposure of FDM 3D-printed composite materials to lubricating oils or industrial fluids can significantly alter their mechanical integrity and microstructural stability. In the case of PLA reinforced with short carbon fibers (PLA+CF), immersion in mineral motor oil may induce physical swelling, softening of the polymer matrix, and changes in fiber–matrix adhesion, depending on the exposure duration and interfacial compatibility.

To evaluate these effects, PLA+CF specimens with a hexagonal infill structure (30% density) were immersed in mineral motor oil for seven days under controlled laboratory conditions. The results were compared with those of unexposed reference specimens to determine the influence of oil exposure on tensile behavior and microstructural integrity.

**Figure 34:**
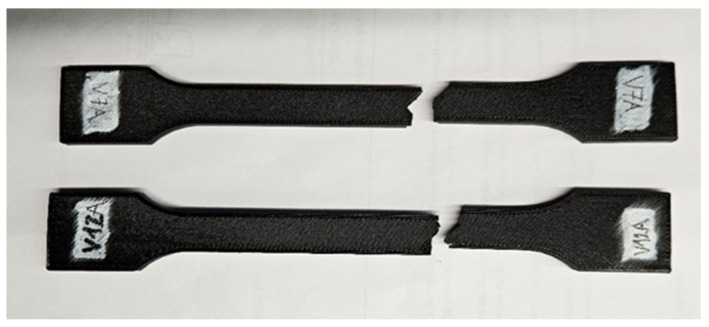
PLA+CF tensile specimens with hexagonal infill (30%): (V7A/PLA+CF_513) unexposed and (V12A/PLA+CF_516) exposed to mineral oil for 7 days. Surface softening and darkening are visible after exposure.

Figure 34 presents the visual comparison between unexposed (V7A/PLA+CF_513) and oil-exposed (V12A/PLA+CF_516) PLA+CF tensile specimens after testing. The oil-exposed samples exhibited a slightly darker surface tone and smoother fracture regions, indicating possible surface plasticization of the matrix.

**Figure 35:**
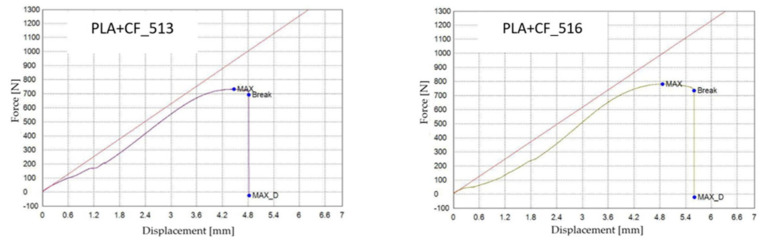
3D-printed PLA+CF specimens tested: the force–displacement curves. The specimens PLA+CF_513 and PLA+CF_516 are representative samples of case V7A and V12A, respectively.

**Figure 36:**
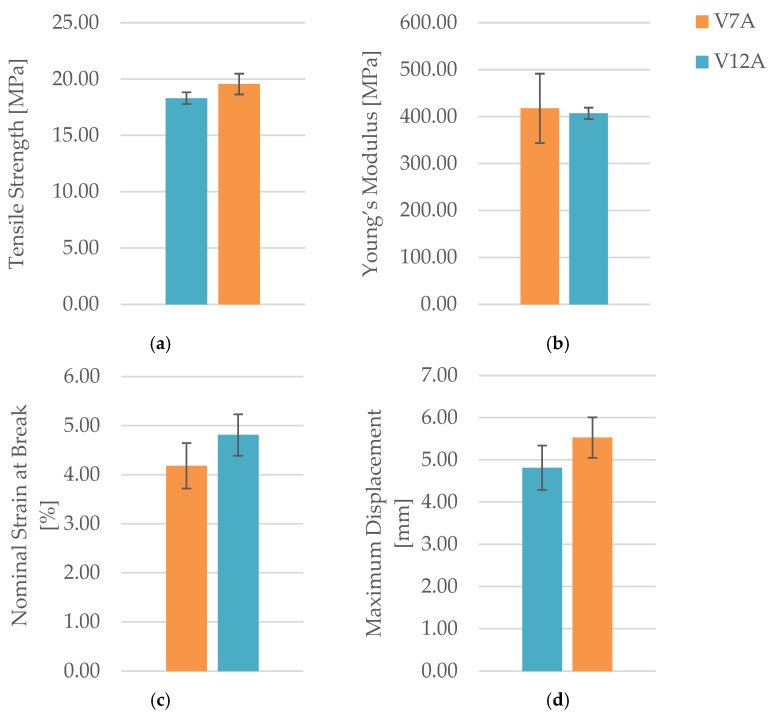
Comparison of tensile properties of PLA+CF specimens with hexagonal infill (30%): (**a**) tensile strength, (**b**) Young’s modulus, (**c**) nominal strain at break, and (**d**) maximum displacement. Oil exposure resulted in a slight increase in strength and ductility, accompanied by a minor reduction in stiffness.

The tensile test results are summarized in Table 13, and the comparative variation in key mechanical parameters—maximum force (F), tensile strength (σ), Young’s modulus (E), nominal strain at break (εₑ), and maximum displacement (Δl)—is illustrated in Figure 35 and Figure 36.

The results indicate that seven-day exposure to mineral oil did not degrade the mechanical performance of PLA+CF specimens. On the contrary, the tensile strength increased by approximately 6.9% (from 18.30 MPa to 19.56 MPa), while Young’s modulus showed a minor reduction of about 2.5%.

The nominal strain at break increased from 4.18% to 4.81%, and the maximum displacement rose by nearly 15%, indicating that oil exposure slightly enhanced ductility and deformation capacity.

These changes suggest that limited oil absorption caused surface plasticization and interfacial stress relaxation, leading to improved filament cohesion and delayed fracture initiation.

From a physicochemical perspective, the observed changes can be attributed to the limited diffusion of low-molecular-weight hydrocarbon chains from the mineral oil into the amorphous PLA regions. This diffusion reduces intermolecular bonding energy and increases local chain mobility, resulting in mild plasticization of the polymer matrix. However, the presence of carbon fibers restricts this diffusion by acting as physical barriers, thereby maintaining overall structural integrity and preventing excessive swelling.

Figure 35 shows representative force–displacement (F−Δl) curves of FDM 3D-printed PLA+CF specimens with a hexagonal infill pattern at 30% infill density, before and after 7-day oil exposure. The specimens PLA+CF_513 (V7A, unexposed) and PLA+CF_516 (V12A, exposed) are presented as representative samples for each condition. The curves indicate that the oil exposure results in minimal changes in the maximum force and displacement at failure, with both cases showing similar load-bearing capacities. However, the exposed specimens (V12A) exhibit a slight increase in strain at break and displacement, suggesting a slight enhancement in ductility after oil exposure. These differences highlight the impact of the oil exposure on the mechanical response of the PLA+CF specimens.

**Figure 37:**
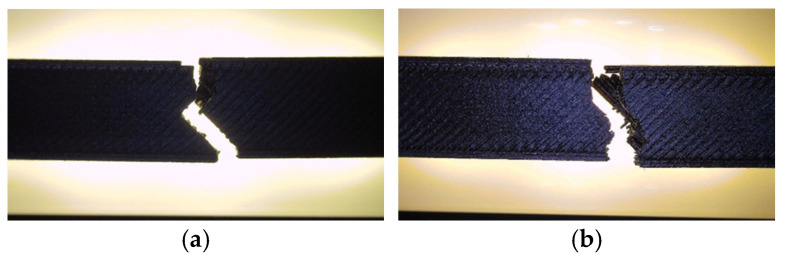
Fracture surfaces of PLA+CF specimens after tensile testing: (**a**) unexposed (V7A) and (**b**) 7-day oil-exposed (V12A). The oil-exposed specimen displays smoother regions and reduced interfacial cracking, consistent with minor matrix softening and improved energy absorption.

Fracture surfaces of the unexposed and oil-exposed specimens are shown in Figure 37. The unexposed PLA+CF sample (V7A) exhibited mixed brittle–ductile fracture zones with visible fiber pull-out and localized shear ridges. After oil exposure, specimen V12A showed smoother fracture surfaces with fewer microvoids and shallower fiber imprints, suggesting improved stress redistribution and reduced crack propagation intensity.

Similar findings were reported by Pascual-González et al. [61] and Guessasma et al. [62], who observed that short-term exposure of FDM composites to non-polar fluids can induce localized chain relaxation without significant degradation. In contrast, prolonged immersion tends to increase interfacial debonding and porosity. The present results confirm that the duration of exposure is a key factor governing the transition between reversible plasticization and irreversible deterioration in PLA-based composites.

These observations support the mechanical results, indicating that moderate oil absorption improved local ductility without causing significant degradation of the fiber–matrix interface.

**Figure 38:**
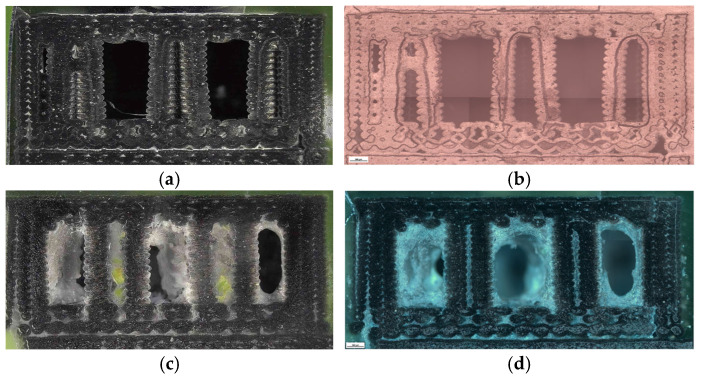
Optical micrographs of unfractured PLA+CF specimens with hexagonal infill (30%): (**a**,**b**) unexposed (V7B) and (**c**,**d**) 7-day oil-exposed (V12B). The exposed sample shows a more uniform but finer pore distribution, consistent with diffusion-induced restructuring of filament interfaces.

Unfractured specimens were examined under optical microscopy to assess internal porosity and filament cohesion (Figure 38).

The unexposed specimen (V7B) displayed compact filament alignment and moderately distributed pores.

After oil exposure, the PLA+CF specimen (V12B) exhibited a finer pore structure, with a higher number of smaller voids distributed more uniformly across the matrix.

This transformation indicates a reorganization of interfacial regions due to minor swelling and plasticization of the polymer phase.

**Figure 39:**
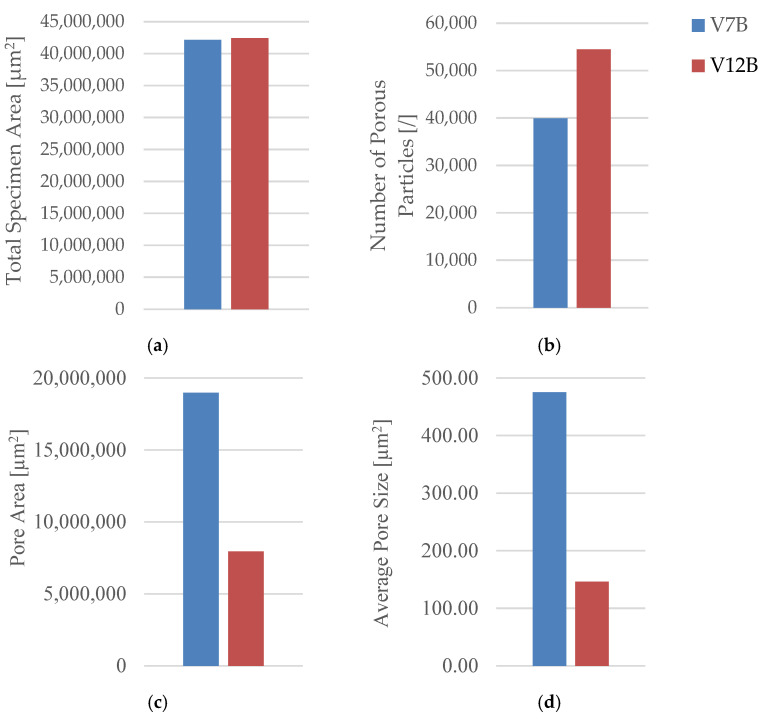
Quantitative microstructural analysis of PLA+CF specimens before and after oil exposure: (**a**) total specimen area, (**b**) number of porous particles, (**c**) pore area, and (**d**) average pore size. Oil exposure increased the number of fine pores but significantly reduced their total area and average size.

Quantitative image analysis (Table 14, Figure 39) confirmed that oil exposure increased the number of detected pores from 39 922 to 54 461, while the total pore area decreased from 18.97 × 10^6^ µm^2^ to 7.95 × 10^6^ µm^2^.

This indicates that smaller voids became more numerous but collectively occupied a smaller area—consistent with pore fragmentation and matrix densification following mild oil-induced swelling.

The quantitative data confirm that short-term oil exposure does not damage the PLA+CF internal structure; instead, it induces redistribution and refinement of pores, improving uniformity and cohesion.

The simultaneous increase in pore count and reduction in total pore area suggests a redistribution of internal stresses at the filament interfaces. This process can be described as a micro-mechanical stabilization mechanism, where smaller, evenly distributed voids act as energy-dissipating zones, delaying crack propagation. The presence of carbon fibers further contributes to this stabilization by constraining local deformation and maintaining load transfer continuity across layers.

The reduction in average pore size from 475 µm^2^ to 146 µm^2^ indicates enhanced filament packing and matrix compactness due to local surface diffusion of polymer chains.

In summary, the seven-day exposure of PLA+CF specimens with hexagonal infill (30%) to mineral oil led to slight strengthening and softening effects occurring simultaneously—a modest increase in tensile strength and ductility, coupled with a small reduction in stiffness.

Microstructural and quantitative analyses confirmed that these changes resulted from fine-scale reorganization of the pore network and partial interfacial plasticization rather than material degradation.

Compared to pure PLA (Section 3.1.3), the PLA+CF composite exhibited higher dimensional and mechanical stability, reflecting the stabilizing effect of carbon fibers, which limit swelling and maintain load transfer capability.

From a thermodynamic standpoint, the diffusion behavior observed can be described by a Fickian-type mechanism limited to the near-surface amorphous zones. The low polarity and high viscosity of mineral oil hinder deep penetration, which explains why plasticization remained localized and reversible. The embedded carbon fibers acted as diffusion barriers, contributing to structural stabilization and maintaining load-bearing continuity throughout the specimen.

These findings are particularly relevant for additive manufacturing of composite components designed for operation in tribological or lubricated environments, such as sealing elements, gear housings, and structural supports in mechanical assemblies. The demonstrated oil resistance of PLA+CF composites highlights their potential as lightweight, dimensionally stable alternatives to conventional thermoplastics in semi-lubricated or maintenance-limited systems. Future investigations should extend to long-term and cyclic exposure tests to establish degradation kinetics and validate performance under dynamic conditions.

## 4. Discussion

The mechanical and microstructural responses of FDM-printed polymers are governed by two interacting mechanisms: (i) geometric control of stress paths through filament orientation and pore topology, and (ii) material-level reinforcement and plasticization processes. The results confirmed that infill geometry and density exert primary control over load transfer efficiency. For PLA, the linear raster aligned with the tensile axis enabled effective axial stress transmission, whereas triangular cells induced stress concentration at vertex junctions. The non-monotonic strength trend with infill density (highest at 30%) suggests that excessive densification can generate internal constraints and premature cracking.

Carbon-fiber reinforcement fundamentally altered these relationships. The fibers bridged interlayer gaps and constrained polymer mobility, resulting in nearly linear strengthening with increasing infill density. This trend indicates that mechanical integrity in FDM composites depends more on interfacial consolidation than on the nominal material fraction.

It should be emphasized that the maximum tensile strength observed for neat PLA at 30% infill and for PLA+CF at 100% infill arises from fundamentally different load-transfer mechanisms related to matrix-dominated versus fiber-reinforced behavior.

Environmental exposure further highlighted the contrasting behavior of both systems. PLA showed partial softening and micro-porosity refinement consistent with mild plasticization, while PLA+CF exhibited a stabilizing effect where fibers acted as barriers to oil diffusion, preserving load-bearing continuity. Quantitative porosity analysis confirmed that strength correlates inversely with total pore area rather than pore count, reinforcing the role of compactness in enhancing mechanical performance.

These findings align with recent studies on the environmental stability of FDM composites, confirming that short-term fluid exposure can promote microstructural rearrangement without significant degradation. Overall, the interplay between filament architecture, carbon-fiber reinforcement, and environmental conditioning defines the balance between stiffness, strength, and ductility in FDM-printed components. This integrated structure–property framework provides a valuable basis for optimizing the design of polymer–composite parts for semi-lubricated and mechanically demanding applications.

It is important to emphasize that the present results are based on a limited sample size (*n* = 3 per condition), consistent with the ISO 527-2 standard for preliminary comparative tensile testing. Therefore, the obtained mechanical data are intended to represent indicative trends rather than statistically exhaustive outcomes. This approach was adopted deliberately to maintain methodological coherence with our previous research, which already provided statistically validated mechanical characterization of FDM 3D-printed PLA and PLA+CF materials under equivalent process conditions.

In this study, the primary focus was directed toward correlating the mechanical response with microstructural features—such as porosity distribution, interlayer bonding, and morphology—rather than repeating the full statistical evaluation. Nevertheless, a follow-up investigation is already planned to expand the experimental dataset by including a larger number of specimens (*n* ≥ 5–7) and additional environmental parameters (different lubricants, exposure durations, and temperatures). This continuation will enable a more comprehensive statistical validation and further substantiate the preliminary findings presented in this paper.

## 5. Conclusions

This study systematically evaluated the influence of infill geometry, infill density, and short-term mineral oil exposure on the mechanical and microstructural behavior of FDM 3D-printed PLA and PLA+CF composites. The results confirmed that both internal architecture and material composition play decisive roles in defining tensile performance and structural integrity. Triangular and hexagonal infill patterns ensured more uniform stress distribution and improved filament cohesion, while increasing infill density from 30% to 100% markedly enhanced stiffness and strength due to reduced porosity and stronger interlayer bonding.

Carbon-fiber reinforcement further improved dimensional stability, stiffness, and environmental resistance, although with a minor reduction in ductility. After seven days of oil exposure, PLA+CF specimens exhibited mild surface plasticization but maintained or slightly increased tensile strength and ductility, confirming high environmental robustness. Quantitative image analysis showed that smaller and more uniformly distributed pores directly correlate with enhanced load transfer and delayed crack initiation.

Overall, the study demonstrates that the synergistic optimization of printing parameters and material reinforcement substantially improves the mechanical reliability and environmental durability of FDM-printed composites. Despite the encouraging short-term results, this study is limited to static tensile loading and a seven-day exposure period. Future research should therefore address long-term and cyclic environmental effects, including temperature fluctuations and repeated lubrication–drying cycles, to establish predictive models of degradation kinetics. Expanding the methodology to dynamic or fatigue testing would further elucidate the durability of PLA-based composites in operational environments. These findings support the application of PLA+CF in lightweight structural and tribological components operating in semi-lubricated or maintenance-limited environments and provide a foundation for future research on long-term degradation mechanisms and multi-environmental exposure effects.

Future studies will extend the analysis to prolonged exposure and different lubricant types to assess diffusion kinetics and long-term mechanical stability.

## Figures and Tables

**Table 1:** PLA and PLA+CF filaments specifications [72].

Parameter/Material	PLA	PLA+CF
Diameter (mm)	1.75	1.75
Net filament weight (g)	1000	1000
Water absorption (equilibrium in water, 23 °C)	<0.3	0.5
Printing speed (mm/s)	40–60	60–90
Layer height (mm)	0.1–0.2	0.1–0.2
Extrusion temperature (°C)	190–220	200–230
Bed platform temperature (°C)	50–55	40–50

**Table 2:** Mechanical parameters of PLA and PLA+CF filaments [72].

Parameter/Material	PLA	PLA+CF
Density (g/cm^3^)	1.25	~1.29
Tensile Strength (MPa)	45–49	40–45
Young’s modulus (MPa)	1000–1100	1100–1300
Elongation at Break (%)	13.5–15.5	11.5–13.5
Heat Deflection Temperature (°C)	53	60

**Table 3:** Mechanical parameters of FDM 3D-printed PLA specimens with different infill geometries.

Case Code	Specimen Code	Tensile Strength σ [MPa]	Young’s Modulus E [MPa]	Nominal Strain at Break $\varepsilon_{e}$ [%]	Maximum Displacement Δl [mm]	Maximum Force *F* [N]
V1A (hexagonal, 30%)	PLA_211	17.102	473.714	4.890	5.620	684.079
	PLA_212	17.003	453.281	5.012	5.762	680.113
	PLA_213	16.815	335.596	5.085	5.841	672.595
*Average*		*16.973*	*420.867*	*4.991*	*5.741*	*678.929*
*St. Dev.*		*0.146*	*74.547*	*0.100*	*0.112*	*5.833*
V2A (triangular, 30%)	PLA_221	16.653	454.095	3.481	4.005	666.126
	PLA_222	16.994	412.694	3.218	3.692	679.779
	PLA_223	14.861	446.562	2.920	3.364	594.441
*Average*		*16.169*	*437.783*	*3.211*	*3.687*	*646.782*
*St. Dev.*		*1.146*	*22.052*	*0.281*	*0.321*	*45.840*
V3A (linear, 30%)	PLA_231	32.784	797.581	5.100	5.862	1311.360
	PLA_232	31.530	458.760	4.780	5.501	1261.210
	PLA_233	31.500	361.673	4.750	5.459	1260.010
*Average*		*31.938*	*410.216*	*4.880*	*5.607*	*1277.530*
*St. Dev.*		*0.733*	*228.853*	*0.190*	*0.222*	*29.307*

**Table 4:** Microstructural parameters of FDM 3D-printed PLA specimens with different infill geometries.

Specimen Code	Total Specimen Area [µm^2^]	Number of Porous Particles [/]	Pore Area [µm^2^]	Average Pore Size [µm^2^]
V1B (hexagonal, 30%)	41,466,253	9841	19,359,581	1967.24
V2B (triangular, 30%)	41,924,682	39,923	23,326,566	5578.05
V3B (linear, 30%)	41,331,666	19,377	20,736,644	1070.17

**Table 5:** Mechanical parameters of FDM 3D-printed PLA specimens with different infill densities.

Case Code	Specimen Code	Tensile Strength σ [MPa]	Young’s Modulus E [MPa]	Nominal Strain at Break $\varepsilon_{e}$ [%]	Maximum Displacement Δl [mm]	Maximum Force *F* [N]
V3A (linear, 30%)	PLA_231	32.784	797.581	5.100	5.862	1311.360
	PLA_232	31.530	458.760	4.780	5.501	1261.210
	PLA_233	31.500	361.673	4.750	5.459	1260.010
*Average*		*31.938*	*410.216*	*4.880*	*5.607*	*1277.530*
*St. Dev.*		*0.733*	*228.853*	*0.190*	*0.222*	*29.307*
V4A (linear, 60%)	PLA_234	14.454	336.075	3.481	4.005	578.173
	PLA_235	14.276	347.472	3.460	4.005	571.023
	PLA_236	11.858	469.287	3.271	3.985	474.318
*Average*		*13.529*	*384.278*	*3.401*	*3.758*	*541.171*
*St. Dev.*		*1.450*	*73.840*	*0.120*	*0.137*	*58.007*
V5A (linear, 100%)	PLA_237	20.198	451.463	4.160	4.789	807.921
	PLA_238	20.017	423.510	5.062	5.821	800.673
	PLA_239	20.794	521.129	4.391	5.051	831.763
*Average*		*20.336*	*465.367*	*4.541*	*5.220*	*813.452*
*St. Dev.*		*0.407*	*50.273*	*0.470*	*0.536*	*16.266*

**Table 6:** Microstructural parameters of FDM 3D-printed PLA specimens with different infill densities.

Specimen Code	Total Specimen Area [µm^2^]	Number of Porous Particles [/]	Pore Area [µm^2^]	Average Pore Size [µm^2^]
V3B (linear, 30%)	41,331,666	19,377	20,736,644	1070.17
V4B (linear, 60%)	40,973,970	6028	13,558,508	2249.26
V5B (linear, 100%)	39,860,980	3395	3,806,213	1589.23

**Table 7:** Mechanical parameters of FDM 3D-printed PLA specimens with hexagonal infill (30%) before and after mineral oil exposure.

Case Code	Specimen Code	Tensile Strength σ [MPa]	Young’s Modulus E [MPa]	Nominal Strain at Break $\varepsilon_{e}$ [%]	Maximum Displacement Δl [mm]	Maximum Force *F* [N]
V1A (hexagonal, 30%, unexposed)	PLA_211	17.102	473.714	4.891	5.620	684.079
	PLA_212	17.003	453.281	5.011	5.762	680.113
	PLA_213	16.815	335.596	5.079	5.841	672.595
*Average*		*16.973*	*420.864*	*4.990*	*5.741*	*678.929*
*St. Dev.*		*0.146*	*74.547*	*0.100*	*0.112*	*5.833*
V6A (hexagonal, 30%, 7 days exposed)	PLA_214	17.054	454.095	7.810	4.789	682.171
	PLA_215	16.904	412.694	8.601	5.821	676.163
	PLA_216	17.025	454.128	7.792	5.051	681.012
*Average*		*16.994*	*440.306*	*8.071*	*5.220*	*679.782*
*St. Dev.*		*0.080*	*23.912*	*0.460*	*0.536*	*3.187*

**Table 8:** Quantitative microstructural parameters of PLA specimens before and after mineral oil exposure.

Specimen Code	Total Specimen Area [µm^2^]	Number of Porous Particles [/]	Pore Area [µm^2^]	Average Pore Size [µm^2^]
V1B (unexposed)	41,466,253	9841	19,359,581	1967.24
V6B (7 days exposed)	41,500,877	25,338	15,272,284	602.74

**Table 9:** Mechanical parameters of FDM 3D-printed PLA+CF specimens with different infill geometries (30% density).

Case Code	Specimen Code	Tensile Strength σ [MPa]	Young’s Modulus E [MPa]	Nominal Strain at Break $\varepsilon_{e}$ [%]	Maximum Displacement Δl [mm]	Maximum Force *F* [N]
V7A (hexagonal, 30%)	PLA+CF_511	18.156	496.449	5.061	5.824	726.247
	PLA+CF_512	19.134	347.645	5.130	5.902	765.363
	PLA+CF_513	18.299	417.481	4.180	4.815	731.961
*Average*		*18.530*	*420.525*	*4.790*	*5.514*	*741.190*
*St. Dev.*		*0.528*	*74.449*	*0.531*	*0.606*	*21.128*
V8A (triangular, 30%)	PLA+CF_521	17.037	357.035	3.411	3.925	681.496
	PLA+CF_522	19.579	381.023	3.550	4.089	783.157
	PLA+CF_523	16.049	434.175	3.171	3.652	641.990
*Average*		*17.555*	*390.744*	*3.381*	*3.889*	*702.214*
*St. Dev.*		*1.821*	*39.478*	*0.190*	*0.221*	*72.828*
V9A (linear, 30%)	PLA+CF_531	16.049	434.175	3.220	3.705	641.990
	PLA+CF_532	15.718	227.840	3.701	4.256	628.734
	PLA+CF_533	16.536	320.040	4.170	4.803	661.469
*Average*		*16.101*	*327.352*	*3.701*	*4.255*	*644.064*
*St. Dev.*		*0.411*	*103.362*	*0.470*	*0.549*	*16.466*

**Table 10:** Quantitative microstructural parameters of FDM 3D-printed PLA+CF specimens with different infill geometries.

**Specimen** **Code**	**Total** **Specimen Area** **[µm^2^]**	**Number of** **Porous Particles** **[/]**	**Pore** **Area** **[µm^2^]**	**Average** **Pore Size** **[µm^2^]**
V7B (hexagonal, 30%)	42,140,091	39,922	18,972,664	475.24
V8B (triangular, 30%)	41,097,286	25,623	14,841,076	579.21
V9B (linear, 30%)	41,866,158	19,955	23,658,650	1185.60

**Table 11:** Mechanical parameters of FDM 3D-printed PLA+CF specimens with different infill densities (linear infill pattern).

Case Code	Specimen Code	Tensile Strength σ [MPa]	Young’s Modulus E [MPa]	Nominal Strain at Break $\varepsilon_{e}$ [%]	Maximum Displacement Δl [mm]	Maximum Force *F* [N]
V9A (linear, 30%)	PLA+CF_531	16.049	434.175	3.220	3.705	641.990
	PLA+CF_532	15.718	227.840	3.701	4.256	628.734
	PLA+CF_533	16.536	320.040	4.170	4.803	661.469
*Average*		*16.101*	*327.352*	*3.701*	*4.255*	*644.064*
*St. Dev.*		*0.411*	*103.362*	*0.470*	*0.549*	*16.466*
V10A (linear, 60%)	PLA+CF_534	15.859	356.613	3.740	3.925	634.344
	PLA+CF_535	16.511	228.125	3.651	4.195	660.452
	PLA+CF_536	19.020	567.746	2.920	3.652	784.802
*Average*		*16.101*	*384.161*	*3.441*	*3.889*	*693.199*
*St. Dev.*		*1.669*	*171.475*	*0.451*	*0.221*	*80.397*
V11A (linear, 100%)	PLA+CF_537	19.369	304.236	4.521	3.705	774.789
	PLA+CF_538	34.319	525.898	4.960	4.256	1372.770
	PLA+CF_539	33.410	499.886	3.801	4.381	1336.400
*Average*		*29.033*	*443.340*	*4.431*	*4.255*	*1161.320*
*St. Dev.*		*8.381*	*121.168*	*0.590*	*0.549*	*335.239*

**Table 12:** Quantitative microstructural parameters of FDM 3D-printed PLA+CF specimens with different infill densities.

Specimen Code	Total Specimen Area [µm^2^]	Number of Porous Particles [/]	Pore Area [µm^2^]	Average Pore Size [µm^2^]
V9B (linear, 30%)	41,866,158	19,955	23,658,650	1185.60
V10B (linear, 60%)	42,195,455	30,242	14,423,157	476.93
V11B (linear, 100%)	42,523,276	22,235	5,714,268	256.99

**Table 13:** Mechanical parameters of FDM 3D-printed PLA+CF specimens with hexagonal infill (30%) before and after 7-day oil exposure.

Case Code	Specimen Code	Tensile Strength σ [MPa]	Young’s Modulus E [MPa]	Nominal Strain at Break $\varepsilon_{e}$ [%]	Maximum Displacement Δl [mm]	Maximum Force *F* [N]
V7A (hexagonal, 30%, unexposed)	PLA+CF_511	18.156	496.449	5.061	5.824	726.247
	PLA+CF_512	19.134	347.645	5.130	5.902	765.363
	PLA+CF_513	18.299	417.481	4.180	4.815	731.961
*Average*		*18.530*	*420.525*	*4.790*	*5.514*	*741.190*
*St. Dev.*		*0.528*	*74.449*	*0.531*	*0.606*	*21.128*
V12A (hexagonal, 30%, 7 days exposed)	PLA+CF_514	18.026	405.056	4.111	4.728	726.247
	PLA+CF_515	19.592	428.230	4.780	5.500	765.363
	PLA+CF_516	19.557	409.954	4.810	5.532	731.961
*Average*		*19.058*	*414.413*	*4.571*	*5.253*	*741.190*
*St. Dev.*		*0.894*	*12.214*	*0.401*	*0.455*	*21.128*

**Table 14:** Quantitative microstructural parameters of FDM 3D-printed PLA+CF specimens before and after 7-day oil exposure.

Specimen Code	Total Specimen Area [µm^2^]	Number of Porous Particles [/]	Pore Area [µm^2^]	Average Pore Size [µm^2^]
V7B (unexposed)	42,140,091	39,922	18,972,664	475.24
V12B (7-day exposed)	42,416,921	54,461	7,952,110	146.02

## Data Availability

The original contributions presented in this study are included in the article. Further inquiries can be directed to the corresponding author.

## References

[1] Hegab H.A. (2016). Design for additive manufacturing of composite materials and potential alloys: A review. Manuf. Rev..

[2] Saleh Alghamdi S., John S., Roy Choudhury N., Dutta N.K. (2021). Additive Manufacturing of Polymer Materials: Progress, Promise and Challenges. Polymers.

[3] Islam M.A., Mobarak M.H., Rimon M.I.H., Al Mahmud M.Z., Ghosh J., Ahmed M.M.S., Hosain N. (2024). Additive manufacturing in polymer research: Advances, synthesis and applications. Polym. Test..

[4] Wickramasinghe S., Do T., Tran P. (2020). FDM-Based 3D Printing of Polymer and Associated Composite: A Review on Mechanical Properties, Defects and Treatments. Polymers.

[5] Jayawardane H., Davies I.J., Gamage J.R., John M., Biswas W.K. (2023). Sustainability perspectives—A review of additive and subtractive manufacturing. Sustain. Manuf. Serv. Econ..

[6] Attaran P.T. (2017). The rise of 3-D printing: The advantages of additive manufacturing over traditional manufacturing. Bus. Horiz..

[7] Zhou L., Miller J., Vezza J., Mayster M., Raffay M., Justice Q., Al Tamimi Z., Hansotte G., Sunkara L.D., Bernat J. (2024). Additive Manufacturing: A Comprehensive Review. Sensors.

[8] Ben Said L., Ayadi B., Alharbi S., Dammak F. (2025). Recent Advances in Additive Manufacturing: A Review of Current Developments and Future Directions. Machines.

[9] ASTM Committee F42 on Additive Manufacturing Technologies (2021). Standard Terminology for Additive Manufacturing—General Principles—Terminology.

[10] (2021). Additive Manufacturing—General Principles—Fundamentals and Vocabulary.

[11] ASTM Additive Manufacturing Center of Excellence (AM CoE) (2022). Additive Manufacturing Sector Overview.

[12] Tofail S.A.M., Koumoulos E.P., Bandyopadhyay A., Bose S., O’Donoghue L., Charitidis C. (2018). Additive manufacturing: Scientific and technological challenges, market uptake and opportunities. Mater. Today.

[13] Mwema F.M., Akinlabi E.T. (2020). Basics of Fused Deposition Modelling (FDM). Fused Deposition Modeling. Springer Briefs in Applied Sciences and Technology.

[14] Gibson I., Rosen D.W., Stucker B. (2010). Introduction and Basic Principles. Additive Manufacturing Technologies: Rapid Prototyping to Direct Digital Manufacturing.

[15] Oleksy M., Dynarowicz K., Aebisher D. (2023). Rapid Prototyping Technologies: 3D Printing Applied in Medicine. Pharmaceutics.

[16] Cano-Vicent A., Tambuwala M.M., Hassan S.S., Barh D., Aljabali A.A.A., Birkett M., Arjunan A., Serrano-Aroca Á. (2021). Fused deposition modelling: Current status, methodology, applications and future prospects. Addit. Manuf..

[17] Iftekar S.F., Aabid A., Amir A., Baig M. (2023). Advancements and Limitations in 3D Printing Materials and Technologies: A Critical Review. Polymers.

[18] Arefin A.M.E., Khatri N.R., Kulkarni N., Egan P.F. (2021). Polymer 3D Printing Review: Materials, Process, and Design Strategies for Medical Applications. Polymers.

[19] Mallikarjuna B., Bhargav P., Hiremath S., Jayachristiyan K.G., Jayanth N. (2025). A review on the melt extrusion-based fused deposition modeling (FDM): Background, materials, process parameters and military applications. Int. J. Interact. Des. Manuf. IJIDeM.

[20] Ma T., Zhang Y., Ruan K., Guo H., He M., Shi X., Guo Y., Kong J., Gu J. (2024). Advances in 3D printing for polymer composites: A review. InfoMat.

[21] Mohamed O.A., Masood S.H., Bhowmik J.L. (2015). Optimization of Fused Deposition Modeling Process Parameters: A Review of Current Research and Future Prospects. Adv. Manuf..

[22] Travieso-Rodriguez J.A., Jerez-Mesa R., Llumà J., Traver-Ramos O., Gomez-Gras G., Roa Rovira J.J. (2019). Mechanical Properties of 3D-Printing Polylactic Acid Parts subjected to Bending Stress and Fatigue Testing. Materials.

[23] Martins R.F., Branco R., Martins M., Macek W., Marciniak Z., Silva R., Trindade D., Moura C., Franco M., Malça C. (2024). Mechanical Properties of Additively Manufactured Polymeric Materials—PLA and PETG—For Biomechanical Applications. Polymers.

[24] Lodi H.D., de Campos M.R., dos Reis A.C. (2025). Mechanical, chemical and biological properties of PLA 3D Printer: A systematic review. Res. Soc. Dev..

[25] Karimi A., Rahmatabadi D., Baghani M. (2024). Various FDM Mechanisms Used in the Fabrication of Continuous-Fiber Reinforced Composites: A Review. Polymers.

[26] Blake B., Mendenhall R., Eslami B. (2025). Balancing Strength and Flexibility: Mechanical Characterization of Carbon Fiber-Reinforced PLA Composites in FDM 3D Printing. J. Manuf. Mater. Process..

[27] Camargo J.C., Machado Á.R., Almeida E.C., Silva E.F.M.S. (2019). Mechanical properties of PLA-graphene filament for FDM 3D printing. Int. J. Adv. Manuf. Technol..

[28] Tekinalp H.L., Kunc V., Velez-Garcia G.M., Duty C.E., Love L.J., Naskar A.K., Blue C.A., Ozcan S. (2014). Highly oriented carbon fiber–polymer composites via additive manufacturing. Compos. Sci. Technol..

[29] Abeykoon C., Sri-Amphorn P., Fernando A. (2020). Optimization of fused deposition modeling parameters for improved PLA and ABS 3D printed structures. Int. J. Lightweight Mater. Manuf..

[30] Sheoran A.J., Kumar H. (2020). Fused Deposition modeling process parameters optimization and effect on mechanical properties and part quality: Review and reflection on present research. Mater. Today Proc..

[31] El Magri A., El Mabrouk K., Vaudreuil S., Chibane H., Touhami M.E. (2020). Optimization of printing parameters for improvement of mechanical and thermal performances of 3D printed poly (ether ether ketone) parts. J. Appl. Polym. Sci..

[32] Plamadiala I., Croitoru C., Pop M.A., Roata I.C. (2025). Enhancing Polylactic Acid (PLA) Performance: A Review of Additives in Fused Deposition Modelling (FDM) Filaments. Polymers.

[33] Fernandez-Vicente M., Calle W., Ferrandiz S., Conejero A. (2016). Effect of Infill Parameters on Tensile Mechanical Behavior in Desktop 3D Printing. 3D Print. Addit. Manuf..

[34] Tanveer M.Q., Haleem A., Suhaib M. (2019). Effect of variable infill density on mechanical behaviour of 3-D printed PLA specimen: An experimental investigation. SN Appl. Sci..

[35] Birosz M.T., Andó M. (2024). Effect of infill pattern scaling on mechanical properties of FDM-printed PLA specimens. Prog. Addit. Manuf..

[36] Kadhum A.H., Al-Zubaidi S., Abdulkareem S.S. (2023). Effect of the Infill Patterns on the Mechanical and Surface Characteristics of 3D Printing of PLA, PLA+ and PETG Materials. ChemEngineering.

[37] Daly M., Tarfaoui M., Bouali M., Bendarma A. (2024). Effects of Infill Density and Pattern on the Tensile Mechanical Behavior of 3D-Printed Glycolyzed PET Reinforced with Carbon-Fiber Composites by the FDM Process. J. Compos. Sci..

[38] Turaka S., Jagannati V., Pappula B., Makgato S. (2024). Impact of infill density on morphology and mechanical properties of 3D printed ABS/CF-ABS composites using design of experiments. Heliyon.

[39] Birosz M.T., Ledenyák D., Andó M. (2022). Effect of FDM infill patterns on mechanical properties. Polym. Test..

[40] Tao Y., Kong F., Li Z., Zhang J., Zhao X., Yin Q., Xing D., Li P. (2021). A review on voids of 3D printed parts by fused filament fabrication. J. Mater. Res. Technol..

[41] Dhakal N., Wang X., Espejo C., Morina A., Emami N. (2023). Impact of processing defects on microstructure, surface quality, and tribological performance in 3D printed polymers. J. Mater. Res. Technol..

[42] Baechle-Clayton M., Loos E., Taheri M., Taheri H. (2022). Failures and Flaws in Fused Deposition Modeling (FDM) Additively Manufactured Polymers and Composites. J. Compos. Sci..

[43] Moradi M., Rezayat M., Rozhbiany F.A.R., Meiabadi S., Casalino G., Shamsborhan M., Bijoy A., Chakkingal S., Lawrence M., Mohammed N. (2023). Correlation between Infill Percentages, Layer Width, and Mechanical Properties in Fused Deposition Modelling of Poly-Lactic Acid 3D Printing. Machines.

[44] Gao G., Xu F., Xu J., Tang G., Liu Z. (2022). A Survey of the Influence of Process Parameters on Mechanical Properties of Fused Deposition Modeling Parts. Micromachines.

[45] Abdullah Z., Ting H.Y., Ali M.A.M., Fauadi M.H.F.M., Kasim M.S., Hambali A., Ghazaly M.M., Handoko F. (2018). The Effect of Layer Thickness and Raster Angles on Tensile Strength and Flexural Strength for Fused Deposition Modeling (FDM) Parts. J. Adv. Manuf. Technol. JAMT.

[46] Chaudhry M.S., Czekanski A. (2020). Evaluating FDM Process Parameter Sensitive Mechanical Performance of Elastomers at Various Strain Rates of Loading. Materials.

[47] Głowacki M., Skórczewska K., Lewandowski K., Szewczykowski P., Mazurkiewicz A. (2023). Effect of Shock-Variable Environmental Temperature and Humidity Conditions on FDM Printed Polymers for Tensile Properties. Polymers.

[48] Lee S., Wee J.-W. (2024). Effect of temperature and relative humidity on hydrolytic degradation of additively manufactured PLA: Characterization and artificial neural network modeling. Polym. Degrad. Stab..

[49] Fang L., Yan Y., Agarwal O., Yao S., Seppala J.E., Kang S.H. (2020). Effects of Environmental Temperature and Humidity on the Geometry and Strength of Polycarbonate Specimens Prepared by Fused Filament Fabrication. Materials.

[50] Lendvai L., Fekete I., Rigotti D., Pegoretti A. (2025). Experimental study on the effect of filament-extrusion rate on the structural, mechanical and thermal properties of material extrusion 3D-printed polylactic acid (PLA) products. Prog. Addit. Manuf..

[51] Barrios J.M., Romero P.E. (2019). Improvement of Surface Roughness and Hydrophobicity in PETG Parts Manufactured via Fused Deposition Modeling (FDM): An Application in 3D Printed Self–Cleaning Parts. Materials.

[52] Rivera-López F., Pavón M.M.L., Correa E.C., Molina M.H. (2024). Effects of Nozzle Temperature on Mechanical Properties of Polylactic Acid Specimens Fabricated by Fused Deposition Modeling. Polymers.

[53] Chacón J.M., Caminero M.A., García-Plaza E., Núñez P.J. (2017). Additive manufacturing of PLA structures by fused deposition modelling: Effect of process parameters on mechanical properties and their optimal selection. Mater. Des..

[54] Rogelj A., Liović D., Hozdić E., Franulović M., Mijović B. (2025). Influence of Cooling Lubricants and Structural Parameters on the Tensile Properties of FFF 3D-Printed PLA and PLA/Carbon Fiber Composites. Polymers.

[55] Popescu D., Zapciu A., Amza C., Baciu F., Marinescu R. (2018). FDM process parameters influence over the mechanical properties of polymer specimens: A review. Polym. Test..

[56] Frunzaverde D., Cojocaru V., Ciubotariu C.-R., Miclosina C.-O., Ardeljan D.D., Ignat E.F., Marginean G. (2022). The Influence of the Printing Temperature and the Filament Color on the Dimensional Accuracy, Tensile Strength, and Friction Performance of FFF-Printed PLA Specimens. Polymers.

[57] Dezaki M.L., Ariffin M.K.A.M. (2020). The Effects of Combined Infill Patterns on Mechanical Properties in FDM Process. Polymers.

[58] Dobrosielska M., Dobrucka R., Brząkalski D., Pajewska-Szmyt M., Kurzydłowski K.J., Przekop R.E. (2024). The Influence of Environmental Factors on the Degradation of PLA/Diatomaceous Earth Composites. Polymers.

[59] Hozdić E., Hozdić E. (2023). Comparative Analysis of the Influence of Mineral Engine Oil on the Mechanical Parameters of FDM 3D-Printed PLA, PLA+ CF, PETG, and PETG+CF Materials. Materials.

[60] Hozdić E., Hasanagić R. (2024). Analysis of the Impact of Cooling Lubricants on the Tensile Properties of FDM 3D Printed PLA and PLA+ CF Materials. Polymers.

[61] Pascual-González C., Caraballo J.G.-M., Lizarralde I., Gómez D.G., Fernández-Blázquez J.P. (2024). Additive manufacturing and microstructure effects on thermal and mechanical properties of ply-hybrid carbon and glass fiber composites. Compos. Part B Eng..

[62] Guessasma S., Belhabib S., Altin A. (2020). On the Tensile Behaviour of Bio-Sourced 3D-Printed Structures from a Microstructural Perspective. Polymers.

[63] Özen A., Abali B.E., Völlmecke C., Gerstel J., Auhl D. (2021). Exploring the Role of Manufacturing Parameters on Microstructure and Mechanical Properties in Fused Deposition Modeling (FDM) Using PETG. Appl. Compos. Mater..

[64] Shanmugam V., Das O., Babu K., Marimuthu U., Veerasimman A., Johnson D.J., Neisiany R.E., Hedenqvist M.S., Ramakrishna S., Berto F. (2021). Fatigue behaviour of FDM-3D printed polymers, polymeric composites and architected cellular materials. Int. J. Fatigue.

[65] Pandzic A., Hodzic D., Milovanovic A. (2019). Effect of Infill Type and Density on Tensile Properties of PLA Material for FDM Process. Proceedings of the 30th DAAAM International Symposium.

[66] Li T., Song Z., Yang X., Du J. (2023). Influence of Processing Parameters on the Mechanical Properties of Peek Plates by Hot Compression Molding. Materials.

[67] Zhen H., Zhao B., Quan L., Fu J. (2023). Effect of 3D Printing Process Parameters and Heat Treatment Conditions on the Mechanical Properties and Microstructure of PEEK Parts. Polymers.

[68] Patanwala H.S., Hong D., Vora S.R., Bognet B., Ma A.W.K. (2018). The microstructure and mechanical properties of 3D printed carbon nanotube–polylactic acid composites. Polym. Compos..

[69] Naveed N. (2021). Investigating the Material Properties and Microstructural Changes of Fused Filament Fabricated PLA and Tough-PLA Parts. Polymers.

[70] Kumar S., Bhushan P., Sinha N., Prakash O., Bhattacharya S. (2019). Investigation of Structure–Mechanical Property Relationship in Fused Filament Fabrication of Polymer Composites. J. Micromanufactur..

[71] (2012). Plastics—Determination of Tensile Properties—Part 2: Test Conditions for Moulding and Extrusion Plastics.

[72] (2024). Zhejiang Flashforge 3D Technology Co., Ltd., Zhejiang, China. https://www.flashforge.com.

[73] Hozdić E. (2024). Characterization and Comparative Analysis of Mechanical Parameters of FDM- and SLA-Printed ABS Materials. Appl. Sci..

[74] VEVOR Digital Microscope Coin Microscope 10.1in IPS Screen 10-1300X Magnification|VEVOR EU. https://eur.vevor.com/lab-handheld-digital-microscopes-c_12384/vevor-digital-microscope-coin-microscope-10-1in-ips-screen-10-1300x-magnification-p_010710118063.

[75] Carl Zeiss Microscopy, LLC. https://www.micro-shop.zeiss.com/en/us/.

[76] Schindelin J., Arganda-Carreras I., Frise E., Kaynig V., Longair M., Pietzsch T., Preibisch S., Rueden C., Saalfeld S., Schmid B. (2012). Fiji: An open-source platform for biological-image analysis. Nat. Methods.

